# A Model based Survey of Colour Deconvolution in Diagnostic Brightfield Microscopy: Error Estimation and Spectral Consideration

**DOI:** 10.1038/srep12096

**Published:** 2015-07-30

**Authors:** Peter Haub, Tobias Meckel

**Affiliations:** 1 Imaging Consulting, Altlussheim, Germany; 2 Membrane Dynamics, Department of Biology, Technische Universität Darmstadt, Schnittspahnstrasse 3, 64287 Darmstadt, Germany

## Abstract

Colour deconvolution is a method used in diagnostic brightfield microscopy to transform colour images of multiple stained biological samples into images representing the stain concentrations. It is applied by decomposing the absorbance values of stain mixtures into absorbance values of single stains. The method assumes a linear relation between stain concentration and absorbance, which is only valid under monochromatic conditions. Diagnostic applications, in turn, are often performed under polychromatic conditions, for which an accurate deconvolution result cannot be achieved. To show this, we establish a mathematical model to calculate non-monochromatic absorbance values based on imaging equipment typically used in histology and use this simulated data as the ground truth to evaluate the accuracy of colour deconvolution. We show the non-linear characteristics of the absorbance formation and demonstrate how it leads to significant deconvolution errors. In particular, our calculations reveal that polychromatic illumination causes 10-times higher deconvolution errors than sequential monochromatic LED illumination. In conclusion, our model can be used for a quantitative assessment of system components - and also to assess and compare colour deconvolution methods.

Histological or histochemical staining is used to enhance the visual contrast of cell and tissue samples by embedding absorbing dyes into the sample material. To highlight multiple specific cell and tissue structures within a sample, mixtures of multiple stains with different spectral absorption characteristics are deployed. In such stain combinations, however, the contrast information of the individual stains is blurred due to the multiplicative combination of their distinct spectral transmission. Thus, to regain the diagnostic information provided by the contrast of individual stains, reconstruction of the single stain contrast is highly desired.

With the advent of highly automated instrumentation and the trend to automated analysis of histopathology images, a need for sophisticated algorithms arose to decompose optical densities of stain mixtures into stain specific channel information. Several different techniques have been developed over the last years. While Zhou’s^1^ work inspired the application of colour deconvolution (CD) to microscopic images, Ruifrok and Johnston^2^ were first to apply this linear approach to 3-channel colour images. Later, Unsupervised Color Decomposition based on Non-negative Matrix Factorization and Independent Component Analysis^3^ as well as RGB-to-CMYK colour transformation^4^ were developed for the quantification of immunohistochemical (IHC) samples. More recently, a non-linear RGB-to-stain transformation by an artificial neural network approach^5^ and a blind decomposition method with automated estimation of colour vectors^6^ were developed. The latest contributions to the field were made by Chen *et al*.^7^, who achieved a decomposition of more than three stains by an approach based on sparse approximation, and by McCann *et al*.^8^, who improved the linear CD method by geometric colour vector determination.

The approach by Ruifrok and Johnston^2^ forms the de-facto standard for stain separation^3^,^7^. The spatial distribution of the pure stain concentrations are calculated by transforming the red, green, and blue (RGB) channels of colour images yielding ‘stain channels’ which contain the relative stain concentration. The method is well accepted in the scientific community and implemented in many commercial and open source bio-imaging software packages, like ScanScope and ImageJ.

Linear and blind colour deconvolution methods are a frequent part of analysis approaches that are based on quantitative and often automated image analysis procedures. For example, CD has been used to detect and classify subcellular protein patterns^9^. More importantly, however, CD based quantification of IHC tissue microarrays has shown good correlation to manual scoring^10^,^11^ and was applied in fully automated manner to score cell death processes^12^. An open source CD software plugin helped to lower the application barrier so that the method was used in several works to quantify IHC samples^13^,^14^,^15^ and the fact that CD based images were found to achieve the best object segmentation results^16^ may explain its quick adoption and distribution. In addition, CD is of particular importance for the *normalisation* of colour in microscopic images, as reviewed elsewhere^17^. It is used in combination with a colour vector estimation^18^ and as a part of a machine learning approaches^19^ to normalise histological images.

While this broad application spectrum leaves no doubt about the value and usefulness of CD, the method’s theory is based on an assumption, which is not always met by the circumstances of its application. Ruifrok’s CD method is based on the assumption of a linear relation between the spectral absorbance of a stain mixture and the concentrations of the pure stains. While this assumption is consistent with the Lambert-Beer law under monochromatic conditions, its validity is questionable under non-monochromatic conditions, i.e. when deconvolution is applied to colour images captured with RGB colour cameras equipped with spectral band filters as it is commonly done in histopathological imaging. A way to assess the error introduced by the non-monochromatic situations lies in the fully theoretical examination of the deconvolution process based on synthetic images, where absolute and quantitative comparisons can be drawn based on a precisely known ground truth. To our knowledge, this approach, i.e. the theoretical examination of the CD method, has not been undertaken so far.

To change this, we have simulated the physical signal formation by numerical calculations of spectral light absorption as they are caused by biological specimens stained with a mixture of dyes. By cumulating the contributions of spectral stain transmittance, spectral sensor sensitivity and spectral characteristics of wideband (D65, I_rel_ = 1) or narrow band (RGB LED) illumination, we obtain non-monochromatic sensor signals. Based on this benchmark data, we evaluate the CD method by calculating and unmixing absorbance values of stain mixtures.

We use our model (i) to calculate theoretical RGB colour values for varying concentrations of pure stains, (ii) to study the concentration dependency of stain vectors of pure stains, (iii) to calculate the deconvolution error for double and triple staining, (iv) to visualize the non-linear formation of the absorbance values and (v) to demonstrate the effects of the deconvolution error onto cell detection and feature measurement.

In essence, our study intends to characterize the decomposition error, introduced by *linear* deconvolution of *non-linear* data. Quantification of this error is important as inaccuracies created on the CD pre-processing level are likely to propagate into subsequent image analysis procedures, such as object segmentation. Our simulation demonstrates the discrepancy between non-linear signal formation and linear assumption of the CD method. Importantly, our theoretical approach does not comprise error influences from sample preparation and imaging. Therefore, our results describe effects exclusively caused by the methodological error of linear deconvolution of non-linear absorbance signals.

## Material and Methods

### Unmixing and modelling of absorbance values

According to the Bouguer-Lambert-Beer equation the absorption of monochromatic radiation passing absorbing dyes can be described as:


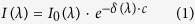


where I_0_(λ) is the spectral radiation intensity, I(λ) is the transmitted spectral intensity, δ(λ) is the spectral molar optical density for a unified layer thickness and c is the dye concentration (see also equation S5).

For a mixture of absorbing stains i a spectral absorbance A(λ) can be expressed as:


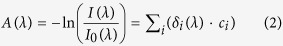


(see also equation S10).

With known spectral absorbance values A_jip_ for different pure stains i a set of linear equations can be formulated to express the spectral absorbance A_j_ for different wavelength j, e.g. for two wavelength and two pure stains as:


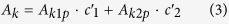



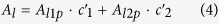


(see also equations S13 and S14).

These linear equations can be used to ‘unmix’ measured absorbance values A_j_ by calculating the relative concentrations c´_1_ and c´_2_. This approach can be extended to higher orders of wavelengths and stains.

A stain vector 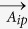 describes the absorbance characteristics of a pure stain i and is expressed e.g. for two monochromatic wavelengths k and l by the stains spectral transmittance τ_p_(λ):


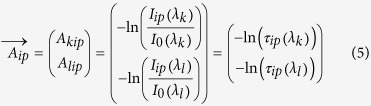


(see also equation S27).

Stain vectors define the target coordinate system for the linear transformation from absorbance into concentration space. Necessarily, they must be specified prior to the deconvolution and can be estimated from samples ideally stained with pure dyes.

If normalised stain vectors with a unit length of 1 are used for unmixing, the resulting normalised relative concentration values c^*^ are related to relative concentration c´ by:


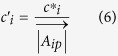


(see also equation S35).

In some diagnostic brightfield applications the absorbance values, used for stain vector estimation and CD, are calculated from the sensor signals V_R_, V_G_, V_B_ measured with scientific RGB colour cameras. For a typical RGB camera with 8 bit maximum colour channel values V_0R_, V_0G_, V_0B_ this can be formulated (without considering any disruptive imaging effects) as:


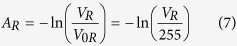



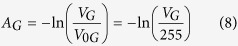



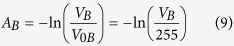


(see also equations S36-S38).

We model the formation of non-monochromatic camera signals V´_R_, V´_G_, V´_B_ by summation of spectral products of light intensity I_rel_(λ), stain transmittance τ_p_(λ), and the sensor characteristics s_R_(λ), s_G_(λ), s_B_(λ). For two stains this can be written as:


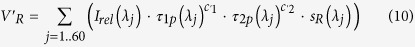



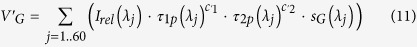



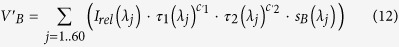


with λ_1_.. λ_60_ = {405 nm, 410 nm, … 700 nm} (see also equations S45-47).

Maximum camera values V´_0R_, V´_0G_, V´_0B_ are calculated without staining (c´_1_ = c´_2_ = 0). Based on these equations the non-linear signal formation is simulated to evaluate the deconvolution of absorbance values derived from these signals.

A detailed description of the theory can be found in Supplementary Information S1 online.

### Spectral Data

All spectral information was available as numerical data sampled with a step size of 5 nm ranging from 405 nm to 700 nm.

**Figure 1:**
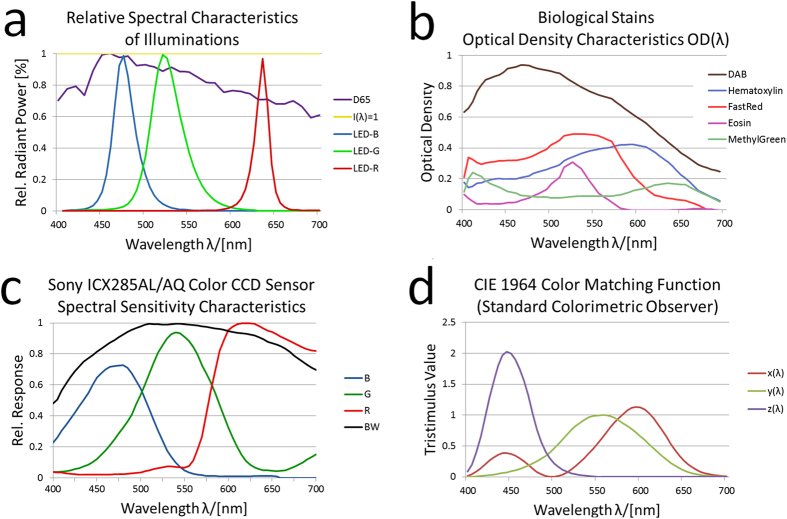
Spectra of illumination, stains, CCD sensors, and CIE tristimulus values. Shown are relative spectral intensities of several illuminations (CIE D65 standard illumination, Cree RGB LED, uniform illumination spectrum I_rel_ = 1) (**a**), optical density spectra of biological stains (**b**), spectral sensitivity characteristics of a Sony ICX285AL/AQ CCD sensor (**c**), and tristimulus functions of the CIE 1964 norm observer (**d**).

The three used illumination spectra (i) CIE D65 standard illumination, (ii) RGB LED illumination and (iii) uniform illumination spectrum (I_rel._(λ) = 1) are shown in Fig. 1a and are derived from the following sources: Spectral data for the D65 standard illumination is specified by the Commission International d’Èclairage [sd-1]. RGB LED spectra were sampled from a Cree® XLamp® XP-E LED data sheet [sd-2] containing spectra for red, green and blue LED types (XPERED, XPEGRN, XPEBLU).

Stain spectra are obtained from CHLA Image Core, Los Angeles [sd-3]. Spectra of hematoxylin (HTX), eosin, diaminobenzidine tetrahydrochloride (DAB), FastRed and MethylenGreen are selected for investigation. The original optical density values are resampled into the 405 nm to 700 nm range in 5 nm step size (Fig. 1b).

From the data sheets of a SONY ICX285AQ Color CCD sensor (Sony, Tokyo, Japan) and a SONY ICX285AL monochrome B/W CCD sensor [sd-4] we sampled the spectral sensitivity of the camera sensor (Fig. 1c). This sensor is commonly used in scientific and industrial applications.

The spectral data of the CIE 1964 standard colorimetric observer (Fig. 1d) was obtained from the tristimulus values specified by the Commission International d’Èclairage [sd-1]. We resampled the data into the 405 nm to 700 nm range in steps of 5 nm.

In particular, we studied the spectral combination of wideband illumination (D65) plus SONY ICX285AQ Color CCD respectively narrow band RGB LED illumination plus SONY ICX285AL monochrome B/W CCD. In the RGB LED/ICX285AL model we suppose a sequential acquisition strategy, whereby a colour image is composed from three monochrome images captured sequentially with red, green and blue LED.

All calculations were performed in Microsoft Excel 2010 (Microsoft, Redmond, WA, USA) and the open source imaging software ImageJ^20^. All data shown are unmodified copies of parts of the Excel tables or ImageJ measurement results.

All error values, indicated in our work, are differences of input and output concentrations. These values do not reflect statistical measurement errors. They are distinct measurement aberrances caused by deficient linear assumption of the CD method. All calculations are performed within the computational precision of 32 bit floating point numbers. Therefore, measurement uncertainties are neglected. Statistical analyses are not applicable to the individual measurement points.

We confirmed the correctness of our mathematical model and our calculations under monochromatic conditions by analysing signal values from distinct wavelengths (see Results in Supplementary Information S1 online). We also showed the relevance and transferability of our model and the used spectral data by a numerical reconstruction of stain colours based on CIE colour calculations (see Fig. S1, S6 and S7 in Supplementary Information S1 online). Additional deconvolution results for double and triple stain models can be found in Tab. S1-S2 and Tab. S3–S6 in Supplementary Information S1 online.

An accompanying ImageJ plugin can be downloaded from http://www.dipsystems.de

## Results and Discussion

### Stain vectors depend on the stain concentration

An essential provision of CD is the accurate determination of the stain vectors for all dyes involved in the stain mixture to be deconvolved. The stain vectors define the coefficient matrix of the linear equations (3) and (4), and therefore represent the spectral absorbance characteristics of the mixed stain. Thus, the linear signal transformation that converts stain intensities into dye concentrations fundamentally depends on these reference vectors. In practice, the vectors of pure stains are determined from slides stained with the single dyes, by calculating the negative logarithm of the relative image signal (see equations (7) to (9), 10, 10).

However, as mentioned before, the signal formation that leads to a microscopic image is a non-linear process because of the polychromatic characteristics of illumination and sensor. Consequently, the stain vectors are derived from signals, which depend on stain concentration. But, they are used as linear coefficients in a linear transformation. We therefore started with an evaluation how the non-linear signal formation influences the stain vectors.

**Figure 2:**
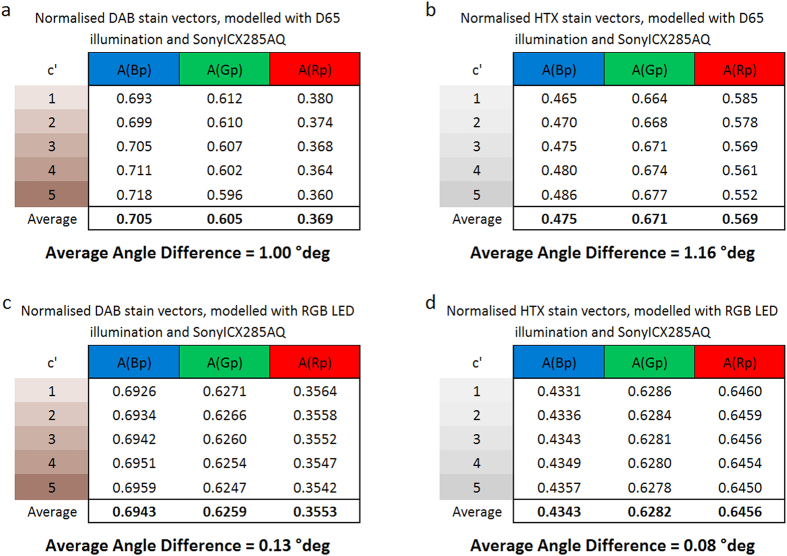
Normalised average stain vectors and average angle variation (AAV), for different concentrations c´_in_, modelled with the combination D65 illumination / Sony ICX285 AQ color CCD sensor for DAB (**a**) and HTX (**b**), and with the combination RGB LED illumination / Sony ICX285 AL monochrome CCD sensor for DAB (**c**) and HTX (**d**).

Stain vectors for pure DAB and pure HTX staining were calculated from modelled camera values. The camera values are computed according to equation (10) to (12), 13, 13 for varying relative stain concentrations (c´ = 1 … 5) and for the different illumination spectra (D65, I_rel_ = 1, RGB LED). The vectors were normalised to unit length (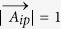). Average stain vectors were calculated and the angles 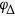, i.e. deviations, between average and normalised vectors, were determined. Finally, the average angle variation (AAV) 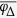 was calculated for each combination of stain and illumination, which can be interpreted as a quality criterion for the stain vector reliability. Results for D65 and RGB LED illumination are shown in Fig. 2.

The AAV for the different illuminations are:


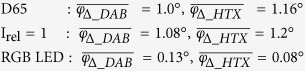


The results demonstrate that the stain vector direction is influenced by the stain concentration – especially for wideband spectral conditions. For narrow band conditions the AAV is ten times smaller than for wideband conditions. Because of the nearly error free stain vectors obtained with the narrow band combination RGB LED/ICX285AL we can call this spectral condition ‘quasi-monochromatic’.

These results show that stain vectors, determined from RGB colour images of purely stained biological samples, are influenced by the image region selected for the vector measurement. Hence, stain vectors cannot be determined distinctly from RGB colour images, in particular not under wideband illumination. In consequence, results of linear deconvolution and the deconvolution error strongly depend on the reference conditions.

For monochromatic conditions, in turn, our numerical evaluation confirms the independency of stain vector directions from illumination spectra and stain concentration. In addition, it confirms that our numerical results comply with the theory (see Methods in Supplementary Information S1 online).

### Spectral conditions strongly influence the error of stain separation

Next, to evaluate the accuracy of stain separation under different illumination conditions, we modelled synthetic images of DAB/HTX double stained slides and deconvolved the non-linear absorbance signals with normalised stain vectors.

The camera signals were modelled according to equation (10) to (12), 13, 13 with wideband D65 and narrow band RGB LED illumination for varying combinations of input concentrations. We calculated the absorbance values (equation (7) to (9), 10, 10) and performed the deconvolution according to equation (3) and (4) with normalised stain vectors from Fig. 2a,b (D65, c´ = 1) and Fig. 2c,d (RGB LED, c´ = 1). The input and output values c*_in_ and c*_out_ are related to these normalised vectors according to equation (6). In this way, both stains equally contribute to the total absorbance. As input values we used c*_in_ = {0.1, 1, 2, 4, 8}.

**Figure 3:**
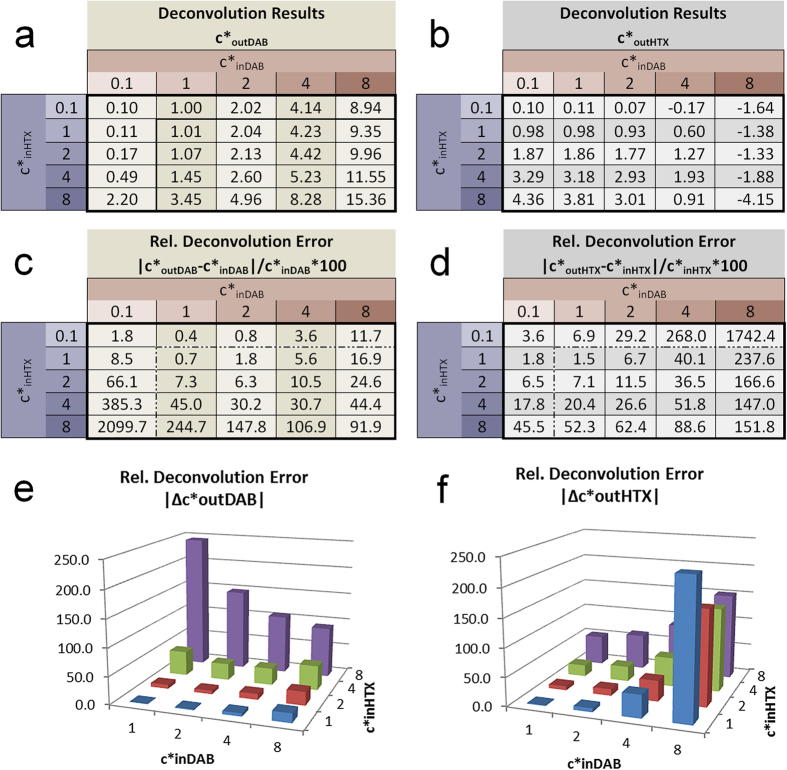
Deconvolution of DAB/HTX double stain modelled with D65 illumination and SonyICX285AQ color CCD sensor. Shown are concentration results c*_out_ for DAB (**a**) and HTX (**b**) and relative deconvolution errors for DAB (**c**,**e**) and HTX (**d**,**f**). The modelling was based on normalised stain vectors and was calculated for different input concentrations c*_in_. The deconvolution was performed in the B/G plane projection.

**Figure 4:**
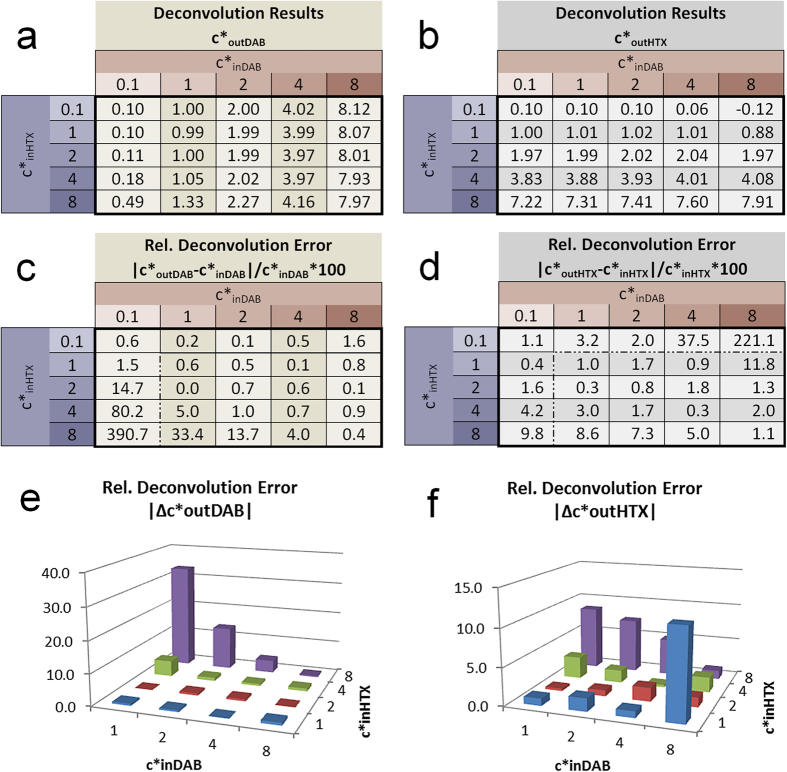
Deconvolution of DAB/HTX double stain modelled with RGB LED illumination and SonyICX285AL monochrome CCD sensor. Shown are concentration results c*_out_ for DAB (**a**) and HTX (b) and relative deconvolution errors for DAB (**c**,**e**) and HTX (**d**,**f**). The modelling was based on normalised stain vectors and was calculated for different input concentrations c*_in_. The deconvolution was performed in the B/G plane projection.

In this double stain model the 3 channels obtained from the RGB camera obviously lead to an over determined system of linear equations. Unmixing is shown here for the B/G plane only (Additional information can be found in Results in Supplementary Information S1 online). The resulting relative concentrations c*_out_ and the relative deconvolution errors Δc^*^_out_ = (c^*^_out_ − c^*^_in_)/c^*^_in_ are shown in Figs. 3 and 4.

The average values of the relative errors |Δ c*_out_| from Fig. 3c,d and Fig. 4c,d for the value range of c*_in_ = {1, 2, 4, 8} are:


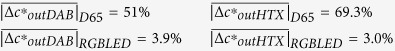


Quite clearly, the error values for the D65 illumination (Fig. 3c,d) are significantly higher than for the RGB LED illumination (Fig. 4c,d). The average D65 deconvolution error for medium and high input concentrations (c*_in_ ≥ 4) exceed 50%. In contrast, the small values obtained under RGB LED illumination again qualifies to consider the light source-camera-combination RGB LED/ICX285AL as ‘quasi-monochromatic’.

Notably, the deconvolution error for one stain increases with the input concentration of the other stain. In other words, errors increase with the difference between stain concentrations. Of particular importance, however, is the finding that all errors reach extreme values for small input concentrations (c*_inDAB_ ~ 0 or c*_inHTX_ ~ 0), a condition that is identical to the unmixing of absorbance values of pure stains. Due to their non-linear signal formation, absorbance values of pure stains are no longer located on the stain vectors and their deconvolution leads to incorrect output signals in both stain channels (see Fig. S4 in Supplementary Information S1 online). This effect clearly illustrates the problem of a linear deconvolution of non-linear signals. Of note, the extreme values for small input concentrations are caused by the small denominator in the calculation of the relative error. They would dominate the average error and are therefore excluded from it.

We found negative concentration values especially for the output concentrations for HTX (c*_outHTX_) under the wideband D65 illumination. This indicates that, due to the non-linear formation, the related absorbance values are located outside of the fans defined by the two stain vectors projected into the absorbance planes. To visualize this, we can imagine two stain vectors spanning a fan in a two dimensional absorbance coordinate system, defining the positive quadrant of a concentration coordinate system. A linear coordinate transformation must then lead to negative concentration values if the absorbance values are located outside of the positive quadrant of this target coordinate system.

The average error values presented above should, however, not be misinterpreted as a general mean deconvolution error. Since the error distribution is extremely non-linear the average values only reflects the error dimension for specific spectral conditions. They rather indicate that the error dimensions are acceptable for a RGB LED illumination, depending on the application requirements, while the error dimensions for the D65 illumination are critical for quantitative applications.

Furthermore, it needs to be kept in mind that the error values for the individual combinations of stain concentrations describe the actual differences between the input and output values and should not be confused with measurement uncertainties. They are the de facto input-output aberrances for specific stain mixtures under specific spectral conditions. Thus, exact error maps could be calculated to correct measurements, if taken under equal spectral conditions. Presumably, the quality of CD results could be improved in this way even if the spectral conditions of the experiment and the error map do not match exactly.

Based on these results, we can draw the conclusions (i) that *under all non-monochromatic system conditions the linear deconvolution results are inaccurate,* (ii) that *full spectrum illumination (like D65 standard illumination) in combination with a wideband sensor lead to significant measurement errors* and (iii) that *sequential narrow band illuminations (like RGB LED illumination) in combination with a wideband sensor lead to acceptable error dimensions*. As a result, the sequential RGB LED illumination can be called ‘quasi-monochromatic’.

Importantly, as our theoretical model assumes ideal experimental conditions, it only uncovers the methodical weakness of linear deconvolution of non-linear signals, while all sources of experimental errors, e.g. from imaging and sample preparation, that would further increase the total errors, are not included.

Of note, we have treated DAB as pure absorber even though it is well known to significantly scatter light in addition to absorbing it, especially at high concentrations. We did this because first, the deconvolution error could as well be studied with arbitrary absorption spectra and second, we intend to determine the undistorted deconvolution error and thereby the maximum achievable accuracy of CD measurements. To avoid misinterpretation, we clearly remind that in fact DAB does not satisfy the Lambert-Beer assumption. Presumably, DAB induces the largest measurement uncertainty besides the sample preparation. As mentioned above, this ‘DAB error’ increases with the concentration–and so does the deconvolution error. A more in-depth comparison of ‘DAB error’ and deconvolution error can be found in a detailed error discussion in Supplementary Information S1 online.

### Deconvolution errors effect cell detection and measurement

**Figure 5:**
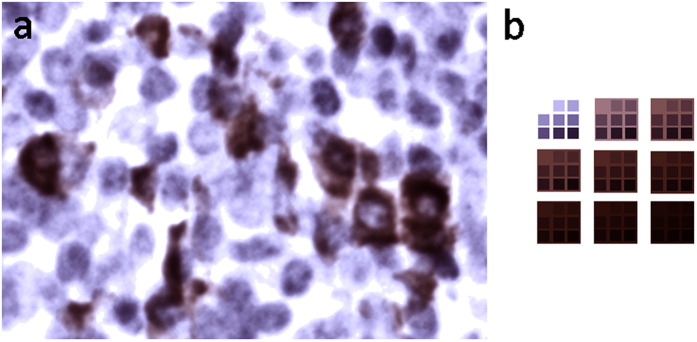
Modelled RGB images, calculated with D65 illumination and Sony ICX285AQ color CCD sensor. Images shown are based on c´_inDAB_ and c´_inHTX_ concentration images mimicking stain distributions of a biological sample (**a**) and on a geometrical arrangement of well-defined c´_in_ values in a self-similar shape (**b**).

To illustrate the deconvolution error under realistic imaging conditions we used DAB and HTX input concentrations to model RGB colour images (example shown in Fig. 5). The input concentrations c´_inDAB_ and c´_inHTX_ are provided as floating point images. The RGB values were calculated pixel-wise for the spectral conditions of D65/ICX285AQ and RGB LED/ICX285AL using the equations (10) to (12), 13, 13.

Subsequently we deconvolved them with non-normalised versions of stain vectors from Fig. 2. The deconvolution was calculated by solving the over-determined system of linear equations with an orthogonalization approach based on the well-known QR decomposition. Lastly, the resulting concentration images c´_outDAB_ and c´_outHTX_ were compared to the input images by calculating the pixel-wise image difference.

**Figure 6:**
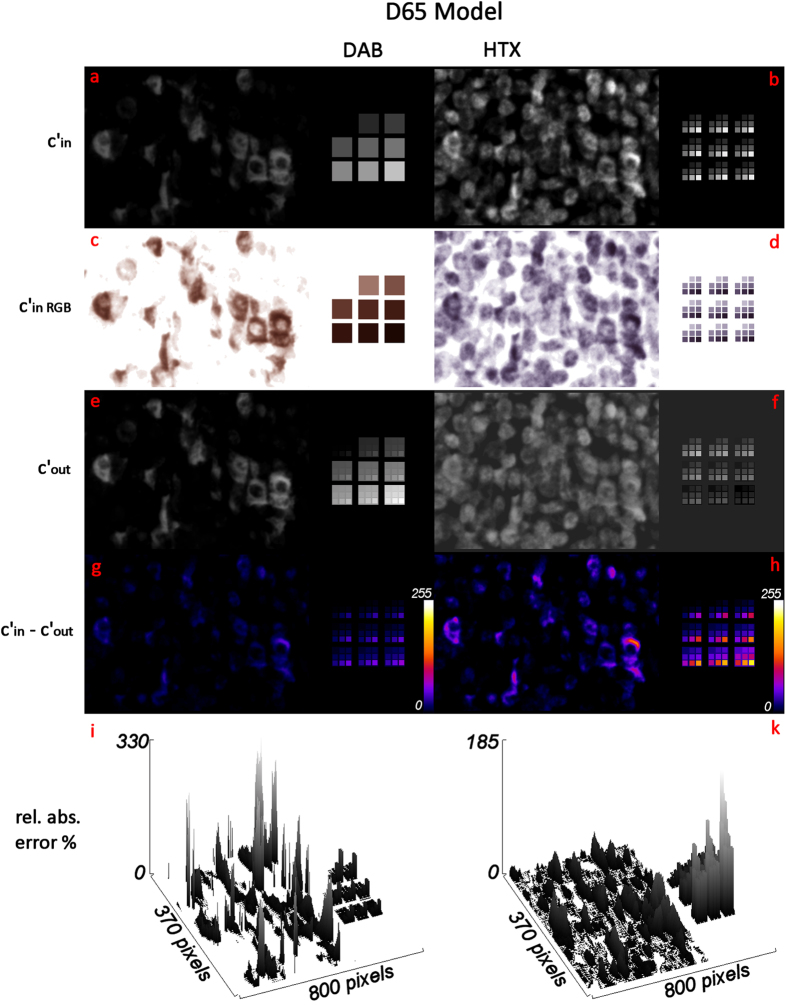
Deconvolution of a modelled DAB/HTX double stain based on D65 illumination/Sony ICX285AQ color CCD sensor (images: c´_in_ in 8 bit grayscale (**a**,**b**) and RGB (**c**,**d**), deconvolution result c´_out_ in 8 bit grayscale (**e**,**f**), difference image c´_in_-c´_out_ in 8 bit with colour LUT (**g**,**h**) and 3D plot of relative absolute error (**i**,**k**)).

**Figure 7:**
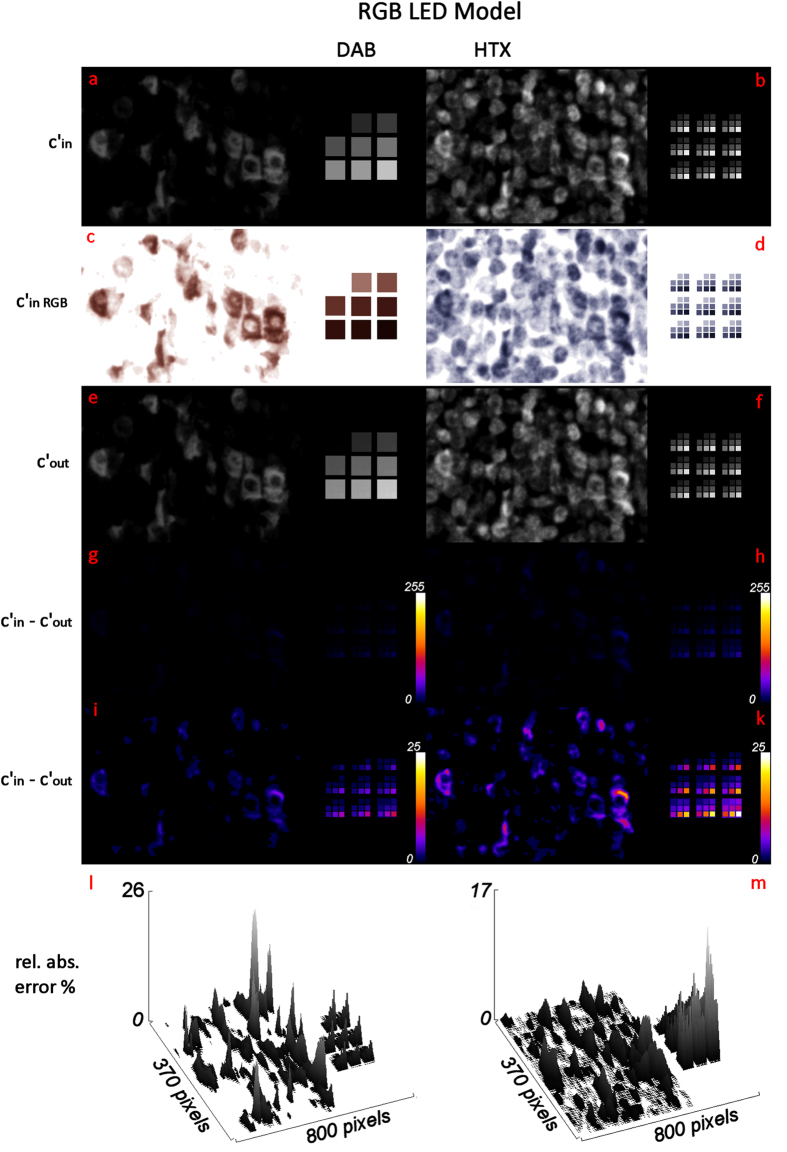
Deconvolution of a modelled DAB/HTX double stain based on RGB LED illumination/Sony ICX285AL monochrome sensor (images: c´_in_ in 8 bit grayscale (**a**,**b**) and RGB (**c**,**d**), deconvolution result c´_out_ in 8 bit grayscale (**e**,**f**), difference image c´_in_-c´_out_ in 8 bit with colour LUT (**g**–**k**) and 3D plot of relative absolute error (**l**,**m**)).

All calculations were carried out with ImageJ^20^ in 32 bit floating point images before scaling them down to 8 bit by a unified maximum display range to allow for direct image comparisons. The minimum display range was set to zero for all grayscale conversions besides for the c´_outHTX_ values obtained with the D65 model, where the minimum value was negative. To visualize the results, input and output concentration images are displayed together with the difference images and relative error plots for the two spectral combinations D65/ICX285AQ (Fig. 6) and RGB LED/ICX285AL (Fig. 7), respectively.

For the D65 model (Fig. 6), the large deconvolution error is directly visible. In the DAB channel of the deconvolution result (c´_outDAB_, Fig. 6e) the brightness of some cell areas is increased compared to the input values used for the colour deconvolution (c´_inDAB_, Fig. 6a). In addition, the tiled modulation in the square areas (i.e. the small squares within large ones, Fig. 6e) reveals a “contamination” of erroneously added values for HTX concentration to the pure DAB test areas. The HTX channel of the deconvolution result (c´_outHTX_, Fig. 6f) shows a decreased cell contrast together with negative concentration values. To visualize this, the background value of c´_outHTX_ = 0 is not shown in black, as for the remaining images, but in dark grey. By calculating the difference between the input and output values for the DAB and HTX values (Fig. 6g,h, respectively) the significant error becomes apparent throughout the image. Plotting the relative error values reveals errors as high as 300% for DAB (Fig. 6i) and nearly 200% for HTX (Fig. 6k).

In stark contrast, the deconvolution error for the LED model (Fig. 7) cannot be observed visually. The difference between c´_in_ and c´_out_ is too small, to be displayed properly in 8-bit images (Fig. 7g,h) and only becomes apparent upon rescaling the difference images to only display the lower 10% of the intensity values (Fig. 7i–k). Likewise, the plots of the relative errors (Fig. 7l,m) show that the maximum relative error is - with about 30% for DAB and 15% for HTX–around 10-times smaller then for the D65 model.

These results confirm all of our prior findings, i.e. (i) the *general deconvolution error*, (ii) the *large error for wideband illumination*, and (iii) the *error acceptable for sequential narrow band illumination*.

Remarkably, both analyses (Figs 6 and 7) demonstrate that the deconvolution error does not alter image contrast to an extent that obscures morphological cell information. All output images appear visually meaningful and therefore the deconvolution error is often overlooked. This is caused by our visual system and should not mislead us underestimate the error. In consequence, however, this means that a visual inspection of CD images is most likely not affected by the deconvolution error, while a computational qualification most probably is.

**Figure 8:**
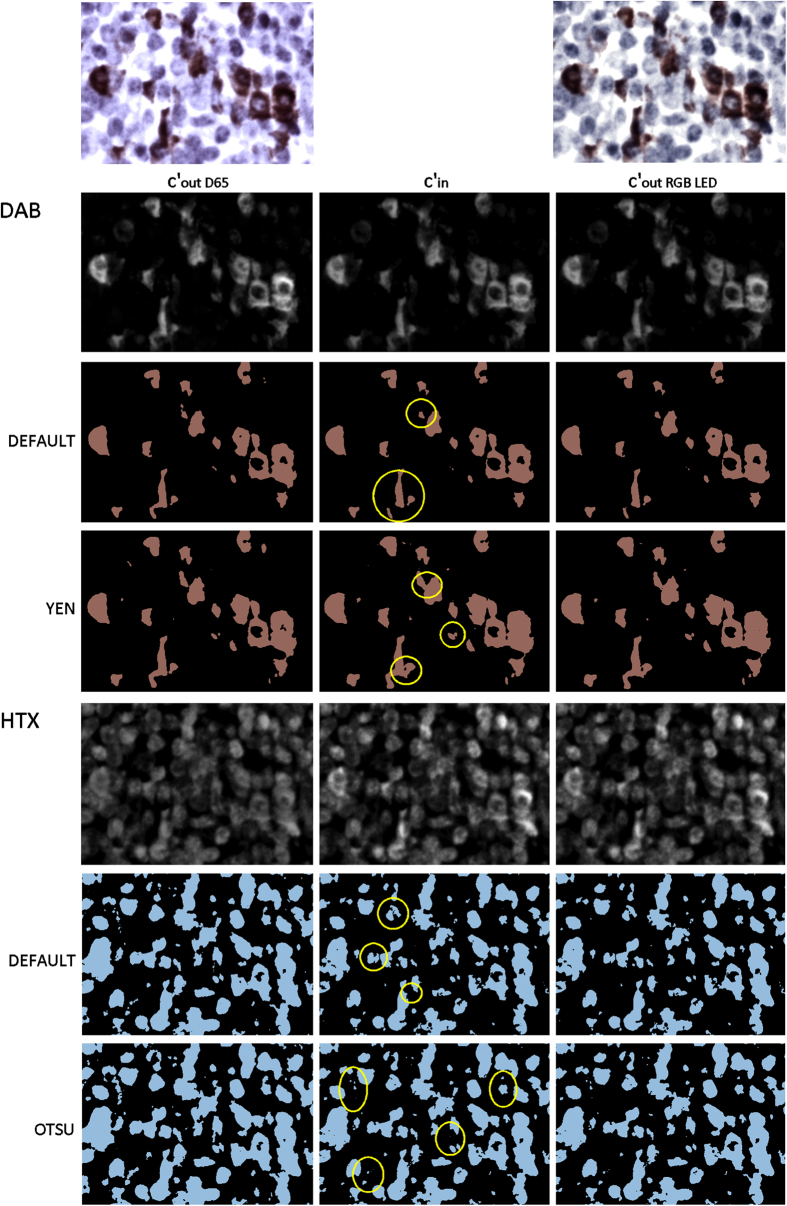
Cell detection in c´_DAB_ and c´_HTX_ concentration images based on different ImageJ automatic threshold modes for input concentration images (middle column) and output concentration images derived by QR deconvolution of RGB images which are modelled with D65 illumination/ICX287AQ (left column) and RGB LED illumination/Sony ICX285AL (right column).

To exemplary analyse the influence of the deconvolution errors for cell qualification and quantification procedures we performed threshold based cell detection. We applied several automatic threshold modes as found in ImageJ, namely ‘Default’, ‘Yen’ and ‘Otsu’, to input and output concentration images. Prior to thresholding, the 32 bit c´_in_ and c´_out_ images were rescaled to 8 bit with a unified minimum and maximum display range. The results (Fig. 8) clearly show that the deconvolution error affects the cell detection. While morphological differences between segmented input and output are recognizable in the D65 data, such differences are absent from the RGB LED data.

**Figure 9:**
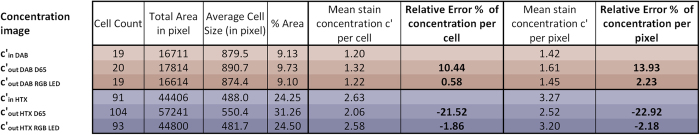
Cell measurements in images of input concentration c´_in_ and deconvolution output c´_out_ for D65/ICX285AQ and RGB LED/ICX285AL model (images segmented with ImageJ threshold method ‘DEFAULT’).

In addition, we quantified the number of cells, cell area and stain concentration per cell and per pixel in the thresholded concentration images. In Fig. 9 results of ImageJ cell measurements are shown for the D65 and RGB LED models. The difference between input and output measurements are considerably higher for the D65 compared to the RGB LED data. Overall, for the D65 model more objects have been detected and the mean object area is increased. The relative errors of the mean cell concentrations and mean pixel concentrations give an idea of the error dimensions. The relative concentration error of individual cells is significant higher than the average error values. For the ten largest cells we found DAB concentrations errors of up to 75% for D65 compared to 7% for RGB LED, with a mean of 28% for D65 respectively 1.9% for RGB LED. Again, all prior findings (i.e. (i) the *general deconvolution error*, (ii) the *large error for wideband illumination*, and (iii) the *error acceptable for sequential narrow band illumination*) are confirmed by this examination.

These error values, however, should not be interpreted as general errors of colour deconvolution or the deconvolution error for a DAB/HTX double staining. They only reflect the conditions of the input concentration c´_inDAB_ and c´_inHTX_ used in this test. In practical situations the error dimensions can differ to higher or lower values, as the actual size of the error depends on the spectral conditions and the concentration distribution of the stains. Nevertheless, our calculation demonstrates that the deconvolution error can have a considerable impact on quantitative image analysis procedures. Additional information can be found in the error discussion in the Supplementary Information S1 online.

## Conclusion

Our study reveals a systematic and methodical error of the CD method under all polychromatic measurement conditions. By using modelled signals we are able to show that the assumption of a linear relation between the spectral absorbance of stains and the stain concentrations only holds true under monochromatic conditions. We demonstrate a concentration dependency of stain vectors, a general deconvolution error under polychromatic conditions and an effect of the deconvolution error onto cell quantification.

The assembly of wideband illumination and detection, as represented in our study by the D65 light source and a colour CCD sensor, lead to significant errors for absorbance signals. Only narrowband illumination and detection, as simulated in our study by RGB LED illumination in combination with a monochrome CCD sensor, a condition that can be seen as ‘quasi-monochromatic’ yields comparably accurate deconvolution results. But even for the latter condition our approach revealed incorrect values, such as negative concentration values and still noticeable deviations between the input and output values. This reminds to a careful interpretation of deconvolution values created under all non-monochromatic system conditions.

Due to the complex signal formation under non-monochromatic conditions the quantification of an overall deconvolution error is not possible. Its dimension depends on the actual system characteristics and stain distributions. A general error for CD cannot be stated. Nevertheless, the presented model allows a valuable estimation of the error dimension for individual spectral conditions. A quantitative assessment can be simply performed by modelling the signal formation with system-specific spectra of the sensor, the illumination and the staining.

Our theoretical approach does not account for any additional error sources or critical system properties such as variations in sample preparation and staining, deviation of stain characteristics from Lambert-Beer behaviour, non-linear electronic characteristics or illumination and imaging effects. Therefore our model represents a best-case scenario and we interpret the demonstrated deconvolution error as the minimal methodical deficiency.

While CD is only a pre-processing step in applications qualifying or quantifying biological structures^9^,^10^,^11^,^12^,^13^,^14^,^15^,^21^, we have shown that deconvolution errors should nevertheless be considered throughout data processing and interpretation. Consequently, results based on CD should be validated against benchmark data, which our method could deliver effortlessly. Finally, the results of our study could provide reliable ground truth data for the development of alternative stain separations approaches^3^,^4^,^5^,^6^,^7^,^8^ and colour normalisation techniques^16^,^17^,^18^.

With our work we intend to help improve photometric analysis of biological stains and not offend the essential CD method. A huge number of studies^1^,^22^,^23^,^24^,^25^ discussed and confirmed the reliability of CD based analysis in the context of application. Indisputable, CD based image analysis has been approved and the correlation of manual scoring and image aided analysis has been shown frequently. CD based image analysis is established in diagnostic pathological.

As a synopsis, we recommend a numerical assessment of the deconvolution error in quantitative diagnostics application and as a result of this, we clearly see a need for the creation of benchmark image data. Furthermore we refer to a potential error reduction by integration of ‘quasi-monochromatic’ hardware such as microscopic RGB LED illumination into microscopic instruments. With the knowledge of illumination spectra and sensor characteristics our approach can lead to new ways of statistical stain spectra estimation from colour images and thereby to new techniques of stain quantification.

## Spectral Data

[sd-1]CIE spectral norm values (Commission International d’Èclairage). Available at https://law.resource.org/pub/us/cfr/ibr/003/cie.15.2004.tables.xls (Accessed: 22nd May 2014).

[sd-2]Cree® XLamp® XP-E LED data sheet. Available at http://www.cree.com/~/media/Files/Cree/LED%20Components%20and%20Modules/XLamp/Data%20and%20Binning/XLampXPE.pdf (Accessed: 06th June 2014).

[sd-3]McNamara, G. 2002. Chromogen Spectra. Los Angeles: CHLA Image Core. Available at http://home.earthlink.net/~geomcnamara/spectra_links.htm (Accessed: 28th May 2014).

[sd-4]Sony ICX285AQ Color CCD sensor data sheet. Available at http://www.sony.net/Products/SC-HP/datasheet/01/data/E01420B3Z.pdf (Accessed: 26th March 2014).

## Additional Information

**How to cite this article**: Haub, P. and Meckel, T. A Model based Survey of Colour Deconvolution in Diagnostic Brightfield Microscopy: Error Estimation and Spectral Consideration. *Sci. Rep*. **5**, 12096; doi: 10.1038/srep12096 (2015).

## Supplementary Material

Supplementary Information

## References

[b1] Zhou R., Hammond E. H. & Parker D. L. A multiple wavelength algorithm in color image analysis and its applications in stain decomposition in microscopy images. Med. Phys. 23, 1977–1986 (1996). 8994162 10.1118/1.597841

[b2] Ruifrok A. C. & Johnston D. A. Quantification of histochemical staining by color deconvolution. Anal. Quant. Cytol. Histol. 23, 291–299 (2001). 11531144

[b3] Rabinovich A., Agarwal S., Laris C., Price J. H. & Belongie S. J. Unsupervised color decomposition of histologically stained tissue samples. Adv. Neural Information Proc. Sys. 16, 667–674 (2003).

[b4] Pham N. A. . Quantitative image analysis of immunohistochemical stains using a CMYK color model. Diagn. Pathol. 2, 8–17 (2007). 17326824 10.1186/1746-1596-2-8 PMC1810239

[b5] Wemmert C. . Stain unmixing in brightfield multiplexed immunohistochemistry. ICIP 13, 1125–1129 (2013).

[b6] Gavrilovic M. . Blind color decomposition of histological images. IEEE Trans. Med. Imaging 32, 983–994 (2013). 23322760 10.1109/TMI.2013.2239655

[b7] Chen T. & Srinivas C. Stain unmixing in brightfield multiplex immunohistochemistry images. (2014). http://stmi2014.ece.cornell.edu/papers/STMI-O-2.pdf (Accessed: 14th October 2014). 10.1016/j.compmedimag.2015.04.001 25920325

[b8] McCann M. T., Majumdar J., Peng C., Castro C. A. & Kovacevic J. Algorithm and benchmark dataset for stain separation in histology images. (2014). http://jelena.ece.cmu.edu/repository/conferences/14_10_ICIP_McCannMPCK.pdf (Accessed: 14th October 2014).

[b9] Newberg J. & Murphy R. F. A framework for the automated analysis of subcellular patterns in human protein atlas images. J. Proteome Res. 7, 2300–2308 (2008). 18435555 10.1021/pr7007626

[b10] Cornish T. C. & Halushka M. K. Color deconvolution for the analysis of tissue microarrays. Anal. Quant. Cytol. Histol. 31, 304–312 (2009). 20701098

[b11] Rizzardi A. E. . Quantitative comparison of immunohistochemical staining measured by digital image analysis versus pathologist visual scoring. Diagn. Pathol. 7, 42–51 (2012). 22515559 10.1186/1746-1596-7-42 PMC3379953

[b12] Krajewska M. . Image analysis algorithms for immunohistochemical assessment of cell death events and fibrosis in tissue sections. J. Histochem Cytochem. 57, 649–663 (2009). 19289554 10.1369/jhc.2009.952812 PMC2699321

[b13] Tuominen V. J., Ruotoistenmaki S., Viitanen A., Jumppanen M. & Isola J. ImmunoRatio: A publicly available web application for quantitative image analysis of estrogen receptor (ER), progesterone receptor (PR), and Ki-67. Breast Cancer Res. 12, R56–67 (2010). 20663194 10.1186/bcr2615 PMC2949645

[b14] Varghese F., Bukhari A. B., Malhotra R. & De A. IHC Profiler: An open source plugin for the quantitative evaluation and automated scoring of immunohistochemistry images of human tissue samples. PloS one 9, e96801 (2014). 24802416 10.1371/journal.pone.0096801 PMC4011881

[b15] Konsti J. . Development and evaluation of a virtual microscopy application for automated assessment of Ki-67 expression in breast cancer. BMC Clin. Patho. 11, 3–13 (2011). 10.1186/1472-6890-11-3 PMC3040126 21262004

[b16] Korzynska A. . Validation of various adaptive threshold methods of segmentation applied to follicular lymphoma digital images stained with 3, 3-diaminobenzidine&haematoxylin. Diagn. Pathol. 8, 48–68 (2013). 23531405 10.1186/1746-1596-8-48 PMC3656801

[b17] Magee D. . Colour normalisation in digital histopathology images. In Proc. Optical Tissue Image analysis in Microscopy, Histopathology and Endoscopy (MICCAI Workshop) 100–111 (2009).

[b18] Macenko M. . A method for normalizing histology slides for quantitative analysis. ISBI 9, 1107–1110 (2009).

[b19] Khan A. M., Rajpoot N., Treanor D. & Magee D. A non-linear mapping approach to stain normalisation in digital histopathology images using image-specific colour deconvolution. IEEE Trans. Biomed. Eng. 61, 1729–1738 (2014). 24845283 10.1109/TBME.2014.2303294

[b20] Schneider C. A., Rasband W. S. & Eliceiri K. W. NIH Image to ImageJ: 25 years of image analysis. Nat. Methods 9, 671–675 (2012). 22930834 10.1038/nmeth.2089 PMC5554542

[b21] Van der Laak J. A., Pahlplatz M. M., Hanselaar A. G. & de Wilde P. Hue‐saturation‐density (HSD) model for stain recognition in digital images from transmitted light microscopy. Cytometry 39, 275–284 (2000). 10738280

[b22] Bernardo V. . Reproducibility of immunostaining quantification and description of a new digital image processing procedure for quantitative evaluation of immunohistochemistry in pathology. Microsc. Microanal. 15, 353–365 (2009). 19575836 10.1017/S1431927609090710

[b23] Ermert L., Hocke A. C., Duncker H. R., Seeger W. & Ermert M. Comparison of different detection methods in quantitative microdensitometry. Am. J. Pathol. 158, 407–417 (2001). 11159179 10.1016/S0002-9440(10)63984-3 PMC1850311

[b24] De Matos L. L., Trufelli D. C., de Matos M. G. L. & da Silva Pinhal M. A. Immunohistochemistry as an important tool in biomarkers detection and clinical practice. Biomarker Insights 5, 9–20 (2010). 20212918 10.4137/bmi.s2185 PMC2832341

[b25] Di Cataldo S., Ficarra E. & Macii E. Computer-aided techniques for chromogenic immunohistochemistry: status and directions. Comput. Biol. Med. 42, 1012–1025 (2012). 22980752 10.1016/j.compbiomed.2012.08.004

