# Supplementary material for: A Model based Survey of Colour Deconvolution in Diagnostic Brightfield Microscopy: Error Estimation and Spectral Consideration
Source: Sci Rep. 2015 Jul 30;5:12096. doi: 10.1038/srep12096 (PMC4519787; doi:10.1038/srep12096)
Supplement: Supplementary Information [file srep12096-s1.pdf]

# Supplementary Information
to

# A Model based Survey of Colour Deconvolution in Diagnostic Brightfield Microscopy:
# Error Estimation and Spectral Consideration

Peter Haub[1] and Tobias Meckel[2]

[1] Imaging Consulting, Altlussheim, Germany [2] Membrane Dynamics, Department of Biology, Technische Universität Darmstadt, Schnittspahnstrasse 3, 64287 Darmstadt, Germany

SUBJECT AREAS:
COLOUR DECONVOLUTION
STAIN SEPARATION
DIGITAL PATHOLOGY
DIAGNOSTIC MICROSCOPY
IMAGE PROCESSING
BIOLOGICAL STAINING

Correspondence and requests for materials should be addressed to phaub@dipsystems.de or tobias.meckel@me.com

in Scientific Reports (2015)

## Content

**Methods: Theory and Modelling** .... 2
S1.1: Theory of Absorption, Absorbance and Deconvolution .... 2
S1.2: Stain Vector - Relative Concentration - Normalization .... 3
S1.3: Numerical Modelling .... 6
**Results: Additional Studies and Results** .... 9
S1.4: Stain Colours from Modelled CIE Colour Values .... 9
S1.5: Modelled Colour Deconvolution - Double Staining .... 10
S1.6: Stain Vector Plane and non-linear Absorbance - 3D-Visualiziation .... 12
S1.7: Modelled Colour Deconvolution - Triple Staining .... 13
S1.8: Display of Concentration Values and Gamma Correction .... 15
**Error Discussion** .... 17
S1.9: Aspects of IHC Preparation .... 17
S1.10: Imaging Errors .... 17
S1.11: DAB Absorption and Scattering .... 19
S1.12: Simulation of DAB Scattering .... 20
**Appendix** .... 24
References .... 24
Symbols .... 25
Indices .... 26

## Methods: Theory and Modelling

### S1.1: Theory of Absorption, Absorbance and Deconvolution

Degression of intensity of radiation I(λ) passing through transparent materials is a function of material properties α(λ), wavelength λ and layer thickness s and it is proportional to the radiation intensity. Mathematically it can be described by:

$$dI(\lambda)/ds = -a(\lambda)\cdot I(\lambda) \tag{S1}$$

The resulting exponential degression of the intensity I(λ) is formulated in the Bouguer-Lambert-Beer equation:

$$I(\lambda) = I_0(\lambda)\cdot e^{-a(\lambda)\cdot s} \tag{S2}$$

For moderately concentrated solutions or stains the absorption coefficient α(λ) correlates linear with the concentration c. The molar absorption coefficient χ(λ) specifies the 'probability of the light absorption'[S1] in relation to the stain concentration and is declared as:

$$\chi(\lambda) = \frac{a(\lambda)}{c} \tag{S3}$$

For practical reasons we introduce here the molar optical density δ(λ), describing the probability of light absorption in relation to the concentration c for a thickness s:

$$\delta(\lambda) = \chi(\lambda)\cdot s = \frac{a(\lambda)}{c}\cdot s \tag{S4}$$

Eqn. (S2) can be rewritten for a unitary thickness s and with the concentration c of an absorbing solution (or stain):

$$I(\lambda) = I_0(\lambda)\cdot e^{-\delta(\lambda)\cdot c} \tag{S5}$$

With the spectral transmission τ(λ) we also can write:

$$\tau(\lambda) = \frac{I(\lambda)}{I_0(\lambda)} = e^{-\delta(\lambda)\cdot c} \tag{S6}$$

$$\delta(\lambda) = \frac{-\ln(\tau(\lambda))}{c} \tag{S7}$$

Multiple filter layers or mixtures of stains lead to a multiplicative product of the spectral transmittance values. The absorption process can be described with:

$$I(\lambda) = \left(\left(\left(I_0(\lambda)\cdot e^{-\delta_1(\lambda)\cdot c_1}\right)\cdot e^{-\delta_2(\lambda)\cdot c_2}\right)\ldots\right)\cdot e^{-\delta_n(\lambda)\cdot c_n} \tag{S8}$$

$$I(\lambda) = I_0(\lambda)\cdot e^{-\sum_{i=1}^{n}(\delta_i(\lambda)\cdot c_i)} = I_0(\lambda)\cdot\prod_{i=1}^{n}\tau_i(\lambda) \tag{S9}$$

With the definition of the spectral absorbance A(λ) as negative logarithm of the relative intensity $I(\lambda)/I_0(\lambda)$, the linear relation between the absorbance of multiple stains and the concentrations of the pure stains follows:

$$A(\lambda) = -\ln\left(\frac{I(\lambda)}{I_0(\lambda)}\right) = \sum_i(\delta_i(\lambda)\cdot c_i) \tag{S10}$$

In case of a mixture of two stains with the concentrations $c_1$ and $c_2$ Eqn. (S10) can be written for two monochromatic wavelengths k and l:

$$A(\lambda_k) = \delta_1(\lambda_k)\cdot c_1 + \delta_2(\lambda_k)\cdot c_2 \tag{S11}$$

$$A(\lambda_l) = \delta_1(\lambda_l)\cdot c_1 + \delta_2(\lambda_l)\cdot c_2 \tag{S12}$$

or in a simplified indexed notation:

$$A_k = \delta_{k1}\cdot c_1 + \delta_{k2}\cdot c_2 \tag{S13}$$

$$A_l = \delta_{l1}\cdot c_1 + \delta_{l2}\cdot c_2 \tag{S14}$$

This system of linear equation can be solved by:

$$c_1 = \frac{\begin{vmatrix} A_k & \delta_{k2} \\ A_l & \delta_{l2} \end{vmatrix}}{\begin{vmatrix} \delta_{k1} & \delta_{k2} \\ \delta_{l1} & \delta_{l2} \end{vmatrix}} \quad \text{and} \quad c_2 = \frac{\begin{vmatrix} \delta_{k1} & A_k \\ \delta_{l1} & A_l \end{vmatrix}}{\begin{vmatrix} \delta_{k1} & \delta_{k2} \\ \delta_{l1} & \delta_{l2} \end{vmatrix}} \quad . \tag{S15}$$

Eqn. (S15) indicates that for a mixture of stains the concentrations $c_1$ and $c_2$ can be calculated from the absorbance values $A_k$ and $A_l$ measured at two monochromatic wavelengths. This coordinate transformation is called ‘spectral unmixing’, ‘stain separation’ or colour deconvolution.

The stain specific absorption characteristics have to be known as so called stain vectors or colour vectors:

$$\vec{\delta_1} = \begin{pmatrix} \delta_{k1} \\ \delta_{l1} \end{pmatrix} \quad \text{and} \quad \vec{\delta_2} = \begin{pmatrix} \delta_{k2} \\ \delta_{l2} \end{pmatrix} \tag{S16}$$

They define the axis of the target coordinate system.

## S1.2: Stain Vector - Relative Concentration - Normalization

The stain specific colour vectors can be determined by measuring the absorbance of reference samples stained with pure dyes at several monochromatic wavelengths.

The following simple equations describe the absorbance of a reference stain at two monochromatic wavelengths k and l according to Eqn. (S10). (Index p indicates the concentration of the pure stain.)

$$A_{k1p} = \delta_{k1}\cdot c_{1p} \tag{S17}$$

$$A_{l1p} = \delta_{l1}\cdot c_{1p} \tag{S18}$$

According to Eqn. (S16) the stain vectors of two pure stains are:

$$\vec{\delta_1} = \begin{pmatrix} \delta_{k1} \\ \delta_{l1} \end{pmatrix} = \begin{pmatrix} \dfrac{A_{k1p}}{c_{1p}} \\ \dfrac{A_{l1p}}{c_{1p}} \end{pmatrix} \quad \text{and} \quad \vec{\delta_2} = \begin{pmatrix} \delta_{k2} \\ \delta_{l2} \end{pmatrix} = \begin{pmatrix} \dfrac{A_{k2p}}{c_{2p}} \\ \dfrac{A_{l2p}}{c_{2p}} \end{pmatrix} \tag{S19}$$

Since $c_{1p}$ and $c_{2p}$ are usually unknown, the vectors $\vec{\delta_1}$ and $\vec{\delta_2}$ cannot be fully determined and therefore cannot be used to solve the system of linear equations. Instead the measured absorbance of the pure stains $A_{kip}$ and $A_{lip}$ can be used to

solve Eqn. (S15). In this case the results $c_1$ and $c_2$ are free of dimension and can - without normalizing the stain vectors - be interpreted as multiples of the concentrations of the pure stains $c_{1p}$ and $c_{2p}$.

This can be seen by rewriting Eqn. (S13) and (S14):

$$A_k = \delta_{k1} \cdot c_{1p} \cdot \frac{c_1}{c_{1p}} + \delta_{k2} \cdot c_{2p} \cdot \frac{c_2}{c_{2p}} \quad \text{(S20)}$$

$$A_l = \delta_{l1} \cdot c_{1p} \cdot \frac{c_1}{c_{1p}} + \delta_{l2} \cdot c_{2p} \cdot \frac{c_2}{c_{2p}} \quad \text{(S21)}$$

With the relative concentration $c'_1$ and $c'_2$ defined as:

$$c'_1 = \frac{c_1}{c_{1p}} \quad \text{(S22)}$$

$$c'_2 = \frac{c_2}{c_{2p}} \quad \text{(S23)}$$

Eqn. (S20) and (S21) simplifies to:

$$A_k = A_{k1p} \cdot c'_1 + A_{k2p} \cdot c'_2 \quad \text{(S24)}$$

$$A_l = A_{l1p} \cdot c'_1 + A_{l2p} \cdot c'_2 \quad \text{(S25)}$$

The solutions of these linear equations are:

$$c'_1 = \frac{\begin{vmatrix} A_k & A_{k2p} \\ A_l & A_{l2p} \end{vmatrix}}{\begin{vmatrix} A_{k1p} & A_{k2p} \\ A_{l1p} & A_{l2p} \end{vmatrix}} \quad \text{and} \quad c'_2 = \frac{\begin{vmatrix} A_{k1p} & A_k \\ A_{l1p} & A_l \end{vmatrix}}{\begin{vmatrix} A_{k1p} & A_{k2p} \\ A_{l1p} & A_{l2p} \end{vmatrix}} \quad \text{(S26)}$$

With Eqn. (S26) the relative concentration values $c'_1$ and $c'_2$ can be calculated from the absorbance values of double stained samples measured at two monochromatic wavelengths under the assumption of a linear relation of absorbance and stain concentrations.

The stain vectors $\overrightarrow{A_{ip}}$ specify the target system for the linear coordinate transformation. They are given by the measured transmission of pure stained reference samples, for example for two monochromatic wavelengths in form of:

$$\overrightarrow{A_{ip}} = \begin{pmatrix} A_{kip} \\ A_{lip} \end{pmatrix} = \begin{pmatrix} -\ln\left(\frac{I_{ip}(\lambda_k)}{I_0(\lambda_k)}\right) \\ -\ln\left(\frac{I_{ip}(\lambda_l)}{I_0(\lambda_l)}\right) \end{pmatrix} = \begin{pmatrix} -\ln(\tau_{ip}(\lambda_k)) \\ -\ln(\tau_{ip}(\lambda_l)) \end{pmatrix} \quad \text{(S27)}$$

Of note, stain vectors $\overrightarrow{A_{ip}}$ have to be called more precisely relative stain vectors since their length depend on the concentration of the pure stains present in the reference samples.

For a mixture of three stains with the concentrations $c'_1$, $c'_2$ and $c'_3$ the Eqn. (S24) and (S25) can be extended for three monochromatic wavelengths k, l and m:

$$A_k = A_{k1p} \cdot c'_1 + A_{k2p} \cdot c'_2 + A_{k3p} \cdot c'_3 \quad \text{(S28)}$$

$$A_l = A_{l1p} \cdot c'_1 + A_{l2p} \cdot c'_2 + A_{l3p} \cdot c'_3 \quad \text{(S29)}$$

$$A_m = A_{m1p} \cdot c'_1 + A_{m2p} \cdot c'_2 + A_{m3p} \cdot c'_3 \quad \text{(S30)}$$

This system of linear equations can be solved by:

$$c'_1 = \frac{\begin{vmatrix} A_k & A_{k2p} & A_{k3p} \\ A_l & A_{l2p} & A_{l3p} \\ A_m & A_{m2p} & A_{m3p} \end{vmatrix}}{\begin{vmatrix} A_{k1p} & A_{k2p} & A_{k3p} \\ A_{l1p} & A_{l2p} & A_{l3p} \\ A_{m1p} & A_{m2p} & A_{m3p} \end{vmatrix}} \; c'_2 = \frac{\begin{vmatrix} A_{k1p} & A_k & A_{k3p} \\ A_{l1p} & A_l & A_{l3p} \\ A_{m1p} & A_m & A_{m3p} \end{vmatrix}}{\begin{vmatrix} A_{k1p} & A_{k2p} & A_{k3p} \\ A_{l1p} & A_{l2p} & A_{l3p} \\ A_{m1p} & A_{m2p} & A_{m3p} \end{vmatrix}} \; c'_3 = \frac{\begin{vmatrix} A_{k1p} & A_{k2p} & A_k \\ A_{l1p} & A_{l2p} & A_l \\ A_{m1p} & A_{m2p} & A_m \end{vmatrix}}{\begin{vmatrix} A_{k1p} & A_{k2p} & A_{k3p} \\ A_{l1p} & A_{l2p} & A_{l3p} \\ A_{m1p} & A_{m2p} & A_{m3p} \end{vmatrix}} \quad \text{(S31)}$$

A stain vector $\overrightarrow{A_{ip}}$ is given for three monochromatic wavelengths by:

$$\overrightarrow{A_{ip}} = \begin{pmatrix} A_{kip} \\ A_{lip} \\ A_{mip} \end{pmatrix} = \begin{pmatrix} -\ln\left(\frac{I_{ip}(\lambda_k)}{I_0(\lambda_k)}\right) \\ -\ln\left(\frac{I_{ip}(\lambda_l)}{I_0(\lambda_l)}\right) \\ -\ln\left(\frac{I_{ip}(\lambda_m)}{I_0(\lambda_m)}\right) \end{pmatrix} \quad \text{(S32)}$$

The direction of such a stain vector is independent from the absolute concentration of the pure stain, since for any concentration c the spectral transmittance $\tau(\lambda)$ can be expressed by the relative concentration $c'$. $c'$ is a constant factor to all vector components due to the logarithmic definition of the absorbance. This can be seen for a 2-dimensional stain vector:

$$\begin{pmatrix} -\ln\left(\left(\tau_{ip}(\lambda_k)\right)^{c'}\right) \\ -\ln\left(\left(\tau_{ip}(\lambda_l)\right)^{c'}\right) \end{pmatrix} = c' \cdot \begin{pmatrix} A_{kip} \\ A_{lip} \end{pmatrix} = c' \cdot \overrightarrow{A_{ip}} \quad \text{(S33)}$$

This is interesting when normalized stain vectors with a unit length of 1 are used for deconvolution. Even though the vector direction is not influenced by this normalization, the deconvolution results $c^*_1$ and $c^*_2$ obtained with those normalized vectors cannot be directly compared to results $c'_1$ and $c'_2$ obtained with non-normalized stain vectors. The ratios of those concentrations are different:

$$\frac{c^*_1}{c^*_2} \neq \frac{c'_1}{c'_2} \quad \text{(S34)}$$

Since the normalization of stain vectors is a scaling of the axis of the vector plane, the results – obtained with normalized and non-normalized vectors – can simply be converted by diving the concentrations $c^*_1$ and $c^*_2$ by the vectors original length:

$$c'_i = \frac{c^*_i}{\left|\overrightarrow{A_{ip}}\right|} \quad \text{(S35)}$$

Therefore, deconvolution results based on normalized and non-normalized stain vectors are equivalent but not identical.

The linear deconvolution of absorbance values determined from intensity values measured at monochromatic wavelengths delivers bijective results and – without considering effects such as signal background, system non-linearity, stain variability and aberrance from Lambert-Beer assumption – error free measurements.

## S1.3: Numerical Modelling

### RGB Camera Signals - Stain Vectors

In some applications the deconvolution method is applied to image values from non-monochromatic devices such as RGB colour cameras. Doing so, the assumption is made that the absorbance values of red, green and blue spectral bands $A_R$, $A_G$, $A_B$ can be calculated as negative logarithms of the ratio of RGB colour channel values $V_R$, $V_G$, $V_B$ and maximum channel values $V_{0R}$, $V_{0G}$, $V_{0B}$. For an 8bit camera this assumption can – without considering signal background, noise effects, white balancing etc. – be written as:

$$A_R = -\ln\left(\frac{V_R}{V_{0R}}\right) = -\ln\left(\frac{V_R}{255}\right) \tag{S36}$$

$$A_G = -\ln\left(\frac{V_G}{V_{0G}}\right) = -\ln\left(\frac{V_G}{255}\right) \tag{S37}$$

$$A_B = -\ln\left(\frac{V_B}{V_{0B}}\right) = -\ln\left(\frac{V_B}{255}\right) \tag{S38}$$

Because of the non-monochromatic and therefore non-linear formation of the camera values, it is questionable if the linear relation of absorbance and stain concentration from Eqn. (S10) is valid for these absorbance values $A_R$, $A_G$, $A_B$.

To address this question, we simulate the formation of camera signals. Our model comprises the spectral sensitivity functions $s_R(\lambda)$, $s_G(\lambda)$, $s_B(\lambda)$ of a scientific grade CCD camera, the spectral intensity distribution of the illumination $I_0(\lambda)$ and the spectral transmittance $\tau_p(\lambda)$ of pure stains.

We describe the formation of the camera values for a pure stain by the integral equations:

$$V_R = k_{R\_CCD} \cdot \int_{\lambda_1}^{\lambda_2} I_0(\lambda) \cdot \tau_p(\lambda)^{c'} \cdot s_R(\lambda) \cdot d\lambda \tag{S39}$$

$$V_G = k_{G\_CCD} \cdot \int_{\lambda_1}^{\lambda_2} I_0(\lambda) \cdot \tau_p(\lambda)^{c'} \cdot s_G(\lambda) \cdot d\lambda \tag{S40}$$

$$V_B = k_{B\_CCD} \cdot \int_{\lambda_1}^{\lambda_2} I_0(\lambda) \cdot \tau_p(\lambda)^{c'} \cdot s_B(\lambda) \cdot d\lambda \tag{S41}$$

(Commonly for the visible wavelength region: $\lambda_1$ = 380 nm, $\lambda_2$ = 780 nm.)
(In the following we assume $k_{R\_CCD} = k_{G\_CCD} = k_{B\_CCD} = 1/[\text{meter}]$.)

Based on these equations the absorbance values $A_R$, $A_G$, $A_B$ of a pure stain can be modelled by:

$$A_R = -\ln\left(\frac{\int_{\lambda_1}^{\lambda_2} I_0(\lambda) \cdot \tau_p(\lambda)^{c'} \cdot s_R(\lambda) \cdot d\lambda}{\int_{\lambda_1}^{\lambda_2} I_0(\lambda) \cdot s_R(\lambda) \cdot d\lambda}\right) \tag{S42}$$

$$A_G = -\ln\left(\frac{\int_{\lambda_1}^{\lambda_2} I_0(\lambda)\cdot\tau_p(\lambda)^{c'}\cdot s_G(\lambda)\cdot d\lambda}{\int_{\lambda_1}^{\lambda_2} I_0(\lambda)\cdot s_G(\lambda)\cdot d\lambda}\right) \tag{S43}$$

$$A_B = -\ln\left(\frac{\int_{\lambda_1}^{\lambda_2} I_0(\lambda)\cdot\tau_p(\lambda)^{c'}\cdot s_B(\lambda)\cdot d\lambda}{\int_{\lambda_1}^{\lambda_2} I_0(\lambda)\cdot s_B(\lambda)\cdot d\lambda}\right) \tag{S44}$$

According to this model, the absorbance values $A_R$, $A_G$, $A_B$ are not linearly related to the stain concentration as in the monochromatic case. The relative concentration c', as exponentiation of the spectral transmittance $\tau_p(\lambda)$, is no longer a constant factor to the vector components. This implicates that (i) the direction of stain vectors derived from those absorbance values depends on the stain concentration and (ii) the linear deconvolution of those non-linear absorbance values delivers incorrect concentration results.

We evaluate the influence of the relative concentration c' onto the stain vector direction and onto the deconvolution result on the basis of a numerical calculation of the modelled camera signals. Our approximation of the camera values $V'_R$, $V'_G$, $V'_B$ for a mixture of two stains is given by the following summations of products of sampled spectral function values:

$$V'_R = \sum_{j=1..60}\left(I_{rel}(\lambda_j)\cdot\tau_{1p}(\lambda_j)^{c'_1}\cdot\tau_{2p}(\lambda_j)^{c'_2}\cdot s_R(\lambda_j)\right) \tag{S45}$$

$$V'_G = \sum_{j=1..60}\left(I_{rel}(\lambda_j)\cdot\tau_{1p}(\lambda_j)^{c'_1}\cdot\tau_{2p}(\lambda_j)^{c'_2}\cdot s_G(\lambda_j)\right) \tag{S46}$$

$$V'_B = \sum_{j=1..60}\left(I_{rel}(\lambda_j)\cdot\tau_{1p}(\lambda_j)^{c'_1}\cdot\tau_{2p}(\lambda_j)^{c'_2}\cdot s_B(\lambda_j)\right) \tag{S47}$$

with $\lambda_1 .. \lambda_{60}$ = {405 nm, 410 nm, … 700 nm}

Spectral data was available in the range from 405 nm to 700 nm. The upper wavelength limit was defined by the IR cut off of the camera. The 5 nm spectral sampling has been confirmed adequate to demonstrate the proposed error effects.

With equations (S45) to (S47) we calculate camera signals and derive the absorbance values for determination of stain vectors and deconvolution of stain mixtures under different spectral conditions. Modelled stain vectors are determined by absorbance values $A_{Rp}$, $A_{Gp}$, $A_{Bp}$ of pure stains:

$$\overrightarrow{A_p} = \begin{pmatrix} A_{Rp} \\ A_{Gp} \\ A_{Bp} \end{pmatrix} = \begin{pmatrix} -\ln\left(\frac{V'_{Rp}}{V'_{0R}}\right) \\ -\ln\left(\frac{V'_{Gp}}{V'_{0G}}\right) \\ -\ln\left(\frac{V'_{Bp}}{V'_{0B}}\right) \end{pmatrix} \tag{S48}$$

Maximum camera values $V'_{0R}$, $V'_{0G}$, $V'_{0B}$ are calculated without staining $\langle c'_1 = c'_2 = 0\rangle$.

As stated before, we assume an influence of the stain concentration onto the stain vector direction due to the non-linear signal formation. This would be crucial for the stain separation because the deconvolution results would depend on the concentration of the pure stains presented in the reference samples. We can use the gradients of the plane projections of

stain vectors to address this important question. These gradients define the vector direction. They are given by the ratios of vector components:

$$\tan(\alpha)=\frac{|A_{Rp}|}{|A_{Gp}|}, \quad \tan(\beta)=\frac{|A_{Rp}|}{|A_{Bp}|}, \quad \tan(\gamma)=\frac{|A_{Gp}|}{|A_{Bp}|} \tag{S49}$$

If absorbance values of pure stains are linear related to the concentration (see Eqn. S33), these gradients are independent from the concentration and so is the vector direction.

One of these ratios is shown exemplarily in the notation of the modelled camera signals:

$$\frac{A_R}{A_G}=\frac{-\ln\left(\dfrac{\int_{\lambda_1}^{\lambda_2} I_0(\lambda)\cdot\tau_p(\lambda)^{c'}\cdot s_R(\lambda)\cdot d\lambda}{\int_{\lambda_1}^{\lambda_2} I_0(\lambda)\cdot s_R(\lambda)\cdot d\lambda}\right)}{-\ln\left(\dfrac{\int_{\lambda_1}^{\lambda_2} I_0(\lambda)\cdot\tau_p(\lambda)^{c'}\cdot s_G(\lambda)\cdot d\lambda}{\int_{\lambda_1}^{\lambda_2} I_0(\lambda)\cdot s_G(\lambda)\cdot d\lambda}\right)}\overset{?}{=}f(c') \tag{S50}$$

It can be seen from this formula that the vector direction is – depending on the spectral conditions - a function of concentration.

To examine the influence of the concentration onto the vector direction, we compare stain vectors from different relative concentrations c' by normalization, averaging and calculating the angle difference $\varphi_\Delta$ between the normalized stain vectors (induced n) and the average stain vector component (induced a) with the equation:

$$\varphi_\Delta=\cos^{-1}\left(A_{nR}\cdot A_{aR}+A_{nG}\cdot A_{aG}+A_{nB}\cdot A_{aB}\right) \tag{S51}$$

We introduce the average angle variation (AAV) $\overline{\varphi_\Delta}$

$$AAV=\overline{\varphi_\Delta}=\frac{\sum_{i=1}^{n}\varphi_{\Delta i}}{n} \tag{S52}$$

as a rating of the vector stability and an estimation of the dimensions of the non-linear influence. High AAV values are signs of a strong influence of stain concentration onto the vector direction. For monochromatic conditions the AAV disappears.

To examine the deconvolution error we model the non-linear absorbance values of stain mixtures and deconvolved this synthetic data. We compared the deconvolution results $c'_{out}$ to the input values $c'_{in}$ by calculating the relative deconvolution error $\Delta c'_{out} = (c'_{out} - c'_{in}) / c'_{in}$ .

Our modelling can demonstrate the dimensions of deconvolution error but cannot deliver a mean error value in the sense of a general measurement uncertainty because of the extreme non-linear nature of the signal formation and the strong dependency on the spectral system conditions.

### CIE Colour Values

Visual colours of stain mixtures can be modelled by integrating spectral products of light intensity, stain transmittance and sensitivity of a CIE norm observer $\bar{x}(\lambda), \bar{y}(\lambda), \bar{z}(\lambda)$. The CIE XYZ colour values can be described for pure stains by:

$$X = \int_{\lambda_1}^{\lambda_2} I_0(\lambda) \cdot \tau_p(\lambda)^{c'} \cdot \bar{x}(\lambda) \cdot d\lambda \qquad \text{(S53)}$$

$$Y = \int_{\lambda_1}^{\lambda_2} I_0(\lambda) \cdot \tau_p(\lambda)^{c'} \cdot \bar{y}(\lambda) \cdot d\lambda \qquad \text{(S54)}$$

$$Z = \int_{\lambda_1}^{\lambda_2} I_0(\lambda) \cdot \tau_p(\lambda)^{c'} \cdot \bar{z}(\lambda) \cdot d\lambda \qquad \text{(S55)}$$

(For the visible wavelength region it can be assumed: $\lambda_1$ = 380 nm, $\lambda_2$ = 780 nm.)

We have approximated the CIE XYZ values for pure stains by the following sums:

$$X = \sum_{j=1..60} \left( I_{D65}(\lambda_j) \cdot \tau_p(\lambda_j)^{c'} \cdot \bar{x}(\lambda_j) \right) \qquad \text{(S56)}$$

$$Y = \sum_{j=1..60} \left( I_{D65}(\lambda_j) \cdot \tau_p(\lambda_j)^{c'} \cdot \bar{y}(\lambda_j) \right) \qquad \text{(S57)}$$

$$Z = \sum_{j=1..60} \left( I_{D65}(\lambda_j) \cdot \tau_p(\lambda_j)^{c'} \cdot \bar{z}(\lambda_j) \right) \qquad \text{(S58)}$$

with $\lambda_1 .. \lambda_{60}$ = {405 nm, 410 nm, … 700 nm}

To create displayable colour values we have converted the resulting XYZ signals into RGB colour signals and transferred them into non-linear sRGB colour values using a gamma correction[S2].

$$\begin{bmatrix} R \\ G \\ B \end{bmatrix} = \begin{pmatrix} 3.240479 & -1.537150 & -0.498535 \\ -0.969256 & 1.875992 & 0.041556 \\ 0.055648 & -0.204043 & 1.057311 \end{pmatrix} \cdot \begin{bmatrix} X \\ Y \\ Z \end{bmatrix} \qquad \text{(S59)}$$

$$\begin{bmatrix} f_\gamma(R) \\ f_\gamma(G) \\ f_\gamma(B) \end{bmatrix} => \begin{bmatrix} R' \\ G' \\ B' \end{bmatrix} \qquad \text{(S60)}$$

We expect that these sRGB colours - displayed on a monitor - are visually similar to the colours of stained biological samples observed at a microscope. This calculation does not necessarily deliver an exact colour reproduction of stain colours. We interpret this calculation as proof of concept and validation of the used spectral data.

## Results: Additional Studies and Results

### S1.4: Stain Colours from Modelled CIE Colour Values

As proof of concept and as a verification of the stain spectra, we calculated the CIE XYZ colour values according to Eqn. (S56) to (S58), for several stains with various concentrations. The XYZ values were converted into non-linear

sRGB values by Eqn. (S59) and (S60). The values were mapped into the 8bit range by a multiplicative intensity scaling in reference to a white signal ⟨c'=0⟩.

Figure S1 displays the modelled stain colours. They comply with the expected colour impression of biological staining products[S3]. We infer that our numeric stain spectra reasonably reproduce real stain spectra. Of note, even if the deconvolution error could as well be studied with arbitrary transmission spectra, realistic spectra help to increase the comprehensibility and the reliability of this study.

Since our signal modelling is an acceptable mathematical model for the colour simulation, we conclude that it is adequate for the examination of the deconvolution error.

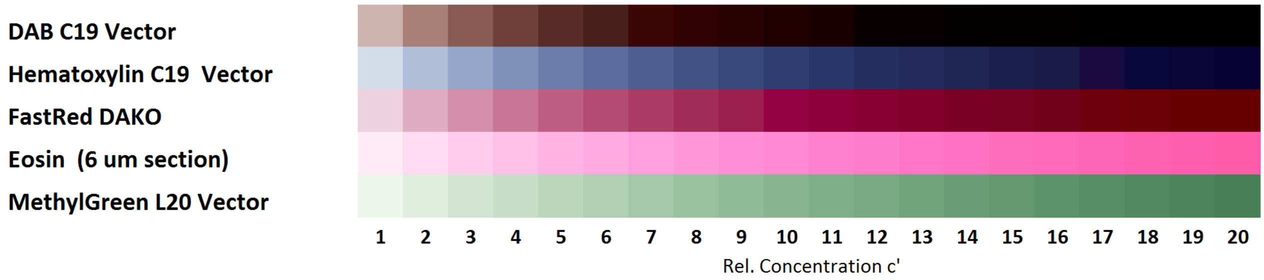


**Supplementary Figure S1 |** ***Modelled sRGB colours*** *of several biological stains for different relative concentration values c' in the range from 1 to 20 (D65 illumination)*

## S1.5: Modelled Colour Deconvolution - Double Staining

To evaluate the deconvolution error, we modelled the camera values for a DAB/HTX double staining with different concentrations $c'_{inDAB}$ and $c'_{inHTX}$ and for different illumination. The camera values were calculated with Eqn. (S45) to (S47). The absorbance values were linearly unmixed with non-normalized stain vectors based on Eqn. (S26). This allows a direct comparison of the input concentrations $c'_{inDAB}$ and $c'_{inHTX}$ and output concentrations $c'_{outDAB}$ and $c'_{outHTX}$. The non-normalized stain vectors were calculated from single stain absorbance modelled with ⟨$c'_{inDAB} = 1$, $c'_{inHTX} = 0$⟩ respectively ⟨$c'_{inDAB} = 0$, $c'_{inHTX} = 1$⟩. Maximum camera values $V'_{0R}$, $V'_{0G}$, $V'_{0B}$ were calculated without staining ⟨$c'_{inDAB} = c'_{inHTX} = 0$⟩.

In Tab. S1 and Tab. S2 the deconvolution results are shown for a few exemplary values of $c'_{inDAB}$ and $c'_{inHTX}$ for the D65 illumination/Sony ICX285AQ and the RGB LED illumination/Sony ICX285AL models. Since the system of linear equations from the 3-dimensional absorbance values is over determined for double staining, the deconvolution can be performed in the three plane projections B/G, B/R, and G/R. The deconvolution results are shown for all three projections.

The results of this modelled DAB/HTX double staining reveal important findings:

A - The deconvolution results $c'_{outDAB}$ and $c'_{outHTX}$ are different in the three plane projections.

This only can be explained if the absorbance values are located outside of the stain vector plane. In this case, the three projections lead to three different systems of linear equations and subsequently to three different pairs of results. This behaviour can be seen especially in the great variance of the deconvolution result modelled with the D65 illumination (Tab. S1).

To eliminate the difference of the result pairs, the absorbance values can be mapped back perpendicular into the vector plane. This leads to identical but not to more exact deconvolution results, since the non-linear absorbance values are not shifted out perpendicular to the vector plane. The exact nature of this shift depends on the spectral characteristics of illumination, stains and sensor. It is rather a complex torsion of the absorbance out of the vector plane (Fig. S4).

Instead of a perpendicular mapping, the over determined system of linear equations can also be solved based on the

Gauss transformation $[c'] = \left([A_p]^T \cdot [A_p]\right)^{-1} \cdot \left([A_p]^T \cdot [A]\right)$ or by an orthogonalization approach, for example with the well-known QR decomposition. We found that the errors are not generally smaller when the deconvolution is based on those methods. For example, the deconvolution errors of the modelled DAB/HTX double stain are minimal for an unmixing in the B/G plane.

B - The results $c'_{outDAB}$ and $c'_{outHTX}$ can become negative especially for wideband illumination spectra. Negative values always occur if absorbance values are lying outside of the fans defined by the stain vector projections.

C - Deconvolution errors emerge under all non-monochromatic system conditions. But the effects are largest for the wideband illumination spectra. With the ‘quasi-monochromatic’ combination of sequential RGB LED illumination and b/w CCD sensor Sony ICX285AL, the error effects still exist but are much less distinct. In this case, the result $c'_{outDAB}$ and $c'_{outHTX}$ are close to the input values $c'_{inDAB}$ and $c'_{inHTX}$ and the differences between the results of the three plane projections are much smaller for the RGB LED illumination.

D - The camera signals were modelled in a monochromatic approach at the three wavelengths (465nm, 540nm, 625nm; specified by the intensity peaks of the RGB LED illumination). A linear deconvolution of these signals delivers always exact results ($c'_{out} = c'_{in}$, within the computational accuracy). The results of the three plane projections are identical and they are independent from the illumination spectra. These findings comply with the theory and confirm the reliability of our model and our calculations.

E - We approved the equivalence of deconvolution with normalized and non-normalized vectors according to Eqn. (S35).

**Supplementary Table S2 | Deconvolution results** $c'_{out}$ for a DAB/HTX double stain modelled with **RGB LED** illumination and non-normalized stain vectors for different input concentrations $c'_{in}$ (deconvolution performed in plane projections B/G, G/R, B/R)

**Non-monochromatic model (signals integrated with Sony ICX285AL spectral sensitivity)**

| | $c'_{inDAB}$=1 $c'_{inHTX}$=1 | | $c'_{inDAB}$=2 $c'_{inHTX}$=1 | | $c'_{inDAB}$=1 $c'_{inHTX}$=2 | | $c'_{inDAB}$=1.25 $c'_{inHTX}$=5 | | $c'_{inDAB}$=5 $c'_{inHTX}$=1.25 | |
|---|---|---|---|---|---|---|---|---|---|---|
| | $c'_{outDAB}$ | $c'_{outHTX}$ | $c'_{outDAB}$ | $c'_{outHTX}$ | $c'_{outDAB}$ | $c'_{outHTX}$ | $c'_{outDAB}$ | $c'_{outHTX}$ | $c'_{outDAB}$ | $c'_{outHTX}$ |
| B/G | 1.00 | 1.02 | 2.00 | 1.01 | 0.99 | 2.03 | 1.25 | 4.99 | 5.04 | 1.08 |
| G/R | 1.01 | 0.97 | 2.02 | 0.94 | 1.03 | 1.94 | 1.34 | 4.76 | 5.05 | 1.04 |
| B/R | 1.00 | 0.99 | 2.01 | 0.96 | 1.01 | 1.97 | 1.29 | 4.83 | 5.04 | 1.05 |

**Monochromatic model (signals sampled at wavelength: 465nm, 540nm, 625nm)**

| | $c'_{inDAB}$=1 $c'_{inHTX}$=1 | | $c'_{inDAB}$=2 $c'_{inHTX}$=1 | | $c'_{inDAB}$=1 $c'_{inHTX}$=2 | | $c'_{inDAB}$=1.25 $c'_{inHTX}$=5 | | $c'_{inDAB}$=5 $c'_{inHTX}$=1.25 | |
|---|---|---|---|---|---|---|---|---|---|---|
| | $c'_{outDAB}$ | $c'_{outHTX}$ | $c'_{outDAB}$ | $c'_{outHTX}$ | $c'_{outDAB}$ | $c'_{outHTX}$ | $c'_{outDAB}$ | $c'_{outHTX}$ | $c'_{outDAB}$ | $c'_{outHTX}$ |
| B/G | 1.00 | 1.00 | 2.00 | 1.00 | 1.00 | 2.00 | 1.25 | 5.00 | 5.00 | 1.25 |
| G/R | 1.00 | 1.00 | 2.00 | 1.00 | 1.00 | 2.00 | 1.25 | 5.00 | 5.00 | 1.25 |
| B/R | 1.00 | 1.00 | 2.00 | 1.00 | 1.00 | 2.00 | 1.25 | 5.00 | 5.00 | 1.25 |

**Supplementary Table S1 | Deconvolution results** $c'_{out}$ for a DAB/HTX double stain modelled with **D65** illumination and non-normalized stain vectors for different input concentrations $c'_{in}$ (deconvolution performed in plane projections B/G, G/R, B/R)

**Non-monochromatic model (signals integrated with Sony ICX285AQ spectral sensitivity)**

| | $c'_{inDAB}$=1 $c'_{inHTX}$=1 | | $c'_{inDAB}$=2 $c'_{inHTX}$=1 | | $c'_{inDAB}$=1 $c'_{inHTX}$=2 | | $c'_{inDAB}$=1.25 $c'_{inHTX}$=5 | | $c'_{inDAB}$=5 $c'_{inHTX}$=1.25 | |
|---|---|---|---|---|---|---|---|---|---|---|
| | $c'_{outDAB}$ | $c'_{outHTX}$ | $c'_{outDAB}$ | $c'_{outHTX}$ | $c'_{outDAB}$ | $c'_{outHTX}$ | $c'_{outDAB}$ | $c'_{outHTX}$ | $c'_{outDAB}$ | $c'_{outHTX}$ |
| B/G | 1.00 | 1.00 | 2.03 | 0.84 | 1.01 | 1.96 | 1.37 | 4.41 | 5.49 | -1.03 |
| G/R | 1.09 | 0.79 | 2.19 | 0.46 | 1.22 | 1.45 | 2.22 | 2.40 | 5.18 | -0.29 |
| B/R | 1.03 | 0.89 | 2.09 | 0.64 | 1.08 | 1.69 | 1.65 | 3.36 | 5.39 | -0.64 |

**Monochromatic model (signals sampled at wavelength: 465nm, 540nm, 625nm)**

| | $c'_{inDAB}$=1 $c'_{inHTX}$=1 | | $c'_{inDAB}$=2 $c'_{inHTX}$=1 | | $c'_{inDAB}$=1 $c'_{inHTX}$=2 | | $c'_{inDAB}$=1.25 $c'_{inHTX}$=5 | | $c'_{inDAB}$=5 $c'_{inHTX}$=1.25 | |
|---|---|---|---|---|---|---|---|---|---|---|
| | $c'_{outDAB}$ | $c'_{outHTX}$ | $c'_{outDAB}$ | $c'_{outHTX}$ | $c'_{outDAB}$ | $c'_{outHTX}$ | $c'_{outDAB}$ | $c'_{outHTX}$ | $c'_{outDAB}$ | $c'_{outHTX}$ |
| B/G | 1.00 | 1.00 | 2.00 | 1.00 | 1.00 | 2.00 | 1.25 | 5.00 | 5.00 | 1.25 |
| G/R | 1.00 | 1.00 | 2.00 | 1.00 | 1.00 | 2.00 | 1.25 | 5.00 | 5.00 | 1.25 |
| B/R | 1.00 | 1.00 | 2.00 | 1.00 | 1.00 | 2.00 | 1.25 | 5.00 | 5.00 | 1.25 |

### S1.6: Stain Vector Plane and non-linear Absorbance - 3D-Visualiziation

To visualize the non-linear behaviour of the signal formation we model a DAB/HTX double staining for the D65 standard illumination. The sensor signals, obtained with Eqn. (S45) to (S47), are converted into absorbance values and then scaled to an 8bit range. The input concentrations $c'_{DAB}$ and $c'_{HTX}$ are varied within the range {0.1, 1, 2, 3, .. 8}. Figure S2 displays the non-linear absorbance values $A_R$, $A_G$, $A_B$ as RGB values in a 3D colour cube.

To visualize the vector plain spanned by the stain vectors, the components of linear absorbance values are calculated following Eqn. (S33). Normalized stain vectors from Fig. 2 (D65, c'=1) (see main document) are used for calculation. Concentrations $c^*_{DAB}$ and $c^*_{HTX}$ are varied within the range {0, 1, 2, .. 10}. Figure S3 displays the linear absorbance values, scaled to 8bit, as RGB values in a 3D colour cube.

In Fig. S4 both data are displayed together. This gives a visual impression of the non-linear formed absorbance in comparison to the linear vector plane of the stain vectors. The non-linear shift and therefore the discrepancy between non-linear absorbance formation and linear assumption are evident.

The different rotations of the 3D cube (Fig. S4) demonstrate that the non-linear absorbance shift is not perpendicular to the vector plane. It is more a rotation out of the fan spanned by the stain vectors. The non-linear absorbance values are drifting out of the vector plane especially for high values of $c'_{DAB}$. This explains the negative $c'_{HTX}$ deconvolution result in Fig. 3 (see main document).

The form of the non-linear shift depends on spectral characteristic of illumination, stains and sensor. It can only be estimated for a particular system setup, with detailed knowledge of its spectral properties.

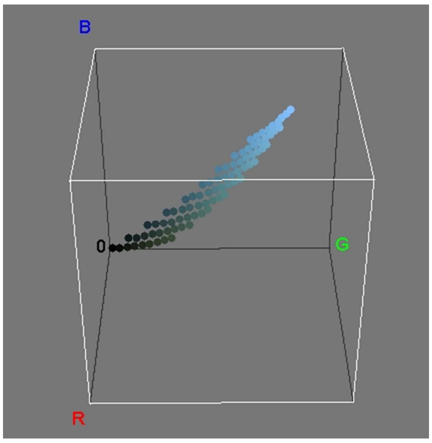


**Supplementary Figure S2 |** ***3D visualization*** *of the modelled* ***non-linear*** *absorbance of a DAB/HTX double staining (coordinate axis $A_R$, $A_G$, $A_B$)*

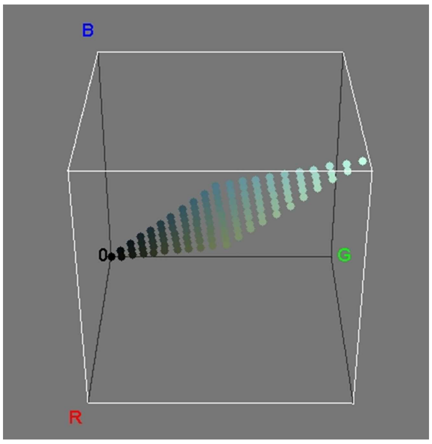


**Supplementary Figure S3 |** ***3D visualization*** *of the* ***vector plane*** *from DAB and HTX stain vectors (coordinate axis $A_R$, $A_G$, $A_B$)*

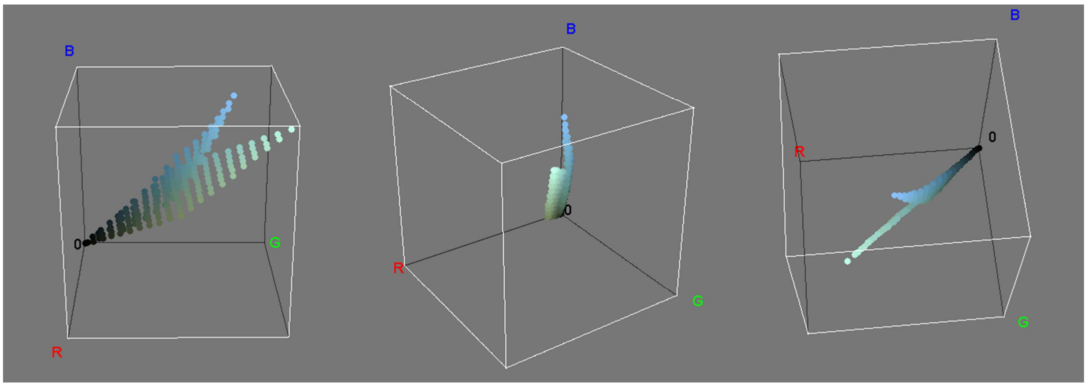


**Supplementary Figure S4 |** ***3D visualization*** *of the combination of stain* ***vector plane and non-linear absorbance*** *of a DAB/HTX double staining. Different rotations of the 3D cube demonstrate the character of the non-linear shift. (coordinate axis $A_R$, $A_G$, $A_B$)*

## S1.7: Modelled Colour Deconvolution - Triple Staining

To analyse the deconvolution error of a triple staining, we compute our model for a stain mixture of DAB, HTX and FastRed. The cameras values are calculated with Eqn. (S45) to (S47), extended by a transmittance variable for the third stain. The relative concentrations of all three stains are varied with $c'_{in} = \{1, 2, 4, 8\}$. The non-normalized stain vectors used for the deconvolution are determined as stated above. The model is calculated for D65 illumination and RGB LED illumination. The deconvolution is performed according to Eqn. (S31).

In Tab. S3 and S5 the 3-dimensional result data are displayed. The table is organized in 4x4 fields for the variations of $c'_{inDAB}$ and $c'_{inHTX}$. Each field exhibits the output values ($c'_{outDAB}$, $c'_{outHTX}$, $c'_{outFastRed}$) for the variation of $c'_{inFastRed}$ (see Fig. S5). The relative errors of $\Delta_{rel.}c'_{out} = (c'_{out} - c'_{in})/c'_{in}$ are shown in the same way in Tab. S4 and S6.

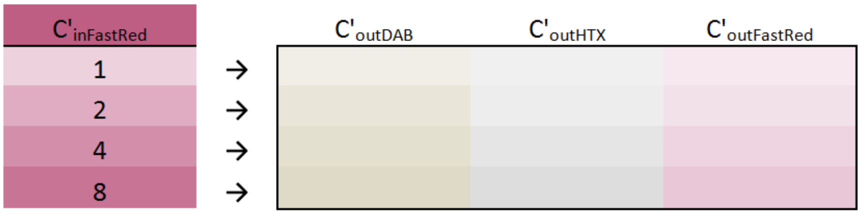


**Supplementary Figure S5 |** ***Result field*** *of the data table: Each field row displays the output values ($c'_{outDAB}$, $c'_{outHTX}$, $c'_{outFastRed}$) for a certain concentration $c'_{inFastRed}$*

According to Tab. S3, DAB dominates the creation of the non-linear absorbance shift. For high concentrations $c'_{inDAB}$ the deconvolution results $c'_{outHTX}$ and $c'_{outFastRed}$ become negative. The absorbance values must be located outside of the volume spanned by the stain vectors. Overall, the values $c'_{in}$ and $c'_{out}$ are not strongly correlated. The data reflects the large non-linearity caused by the wideband D65 illumination.

Deconvolution results of the RGB LED model are more correct (Tab. S5). All result values $c'_{out}$ are close to the input values $c'_{in}$. (Even the logical schema of the result table is reflected by $c'_{out}$.) Only a view negative concentrations values $c'_{out}$ can be found in Tab. S5.

The average absolute errors values $|\Delta c'_{out}|$ from Tab. S4 and Tab. S6 for the value range of $c'_{in} = \{1, 2, 4, 8\}$ are:

$$\overline{|\Delta c'_{outDAB}|}_{D65} = 96.5\% \qquad \overline{|\Delta c'_{outHTX}|}_{D65} = 243.4\% \qquad \overline{|\Delta c'_{outFastRed}|}_{D65} = 146.6\%$$

$$\overline{|\Delta c'_{outDAB}|}_{RGBLED} = 3.9\% \qquad \overline{|\Delta c'_{outHTX}|}_{RGBLED} = 20.9\% \qquad \overline{|\Delta c'_{outFastRed}|}_{RGBLED} = 11.4\%$$

According to Tab. S3-S6 all findings from the double stain models are confirmed by the triple stain model. The relative concentration values $c'_{out}$ are incorrect under all non-monochromatic conditions. The errors are significant larger for D65 illumination compared to RGB LED illumination. While the error dimension of the RGB LED is - depending on the requirements - acceptable for diagnostic applications, almost all D65 error values are in an unacceptable range (see Tab. S4).

**Supplementary Table S3 | Deconvolution results** $c'_{out}$ for a DAB / HTX / FastRed triple stain modelled with **D65** illumination and non-normalized stain vectors for different input concentrations $c'_{in}$. (Rows in fields displays the output values ($c'_{outDAB}$, $c'_{outHTX}$, $c'_{outFastRed}$) for $c'_{inFastRed}$ ={1,2,4,8} (see Fig. S5))

| | $c'_{inDAB}$ = 1 | | | 2 | | | 4 | | | 8 | | |
|---|---|---|---|---|---|---|---|---|---|---|---|---|
| $C'_{inHTX}$ = 1 | 1.00 | 0.75 | 1.15 | 2.08 | 0.40 | 1.16 | 4.55 | -0.79 | 0.60 | 11.12 | -4.81 | -4.04 |
| | 1.09 | 0.47 | 2.10 | 2.25 | -0.01 | 1.98 | 4.98 | -1.49 | 0.93 | 12.00 | -5.90 | -4.71 |
| | 1.51 | -0.43 | 3.54 | 2.92 | -1.20 | 2.96 | 6.23 | -3.24 | 0.78 | 13.82 | -8.08 | -6.30 |
| | 3.75 | -3.60 | 3.70 | 5.70 | -4.88 | 2.04 | 9.77 | -7.57 | -1.85 | 17.21 | -12.08 | -9.63 |
| 2 | 0.98 | 1.54 | 1.31 | 2.07 | 1.10 | 1.37 | 4.63 | -0.31 | 0.72 | 11.54 | -4.79 | -4.47 |
| | 1.09 | 1.19 | 2.25 | 2.27 | 0.61 | 2.13 | 5.13 | -1.10 | 0.93 | 12.47 | -5.94 | -5.23 |
| | 1.59 | 0.14 | 3.60 | 3.06 | -0.74 | 2.95 | 6.53 | -3.02 | 0.52 | 14.38 | -8.24 | -6.96 |
| | 4.06 | -3.35 | 3.38 | 6.08 | -4.73 | 1.61 | 10.27 | -7.62 | -2.45 | 17.90 | -12.43 | -10.44 |
| 4 | 0.98 | 2.86 | 1.72 | 2.11 | 2.23 | 1.78 | 4.92 | 0.39 | 0.80 | 12.46 | -4.91 | -5.46 |
| | 1.15 | 2.37 | 2.58 | 2.41 | 1.58 | 2.39 | 5.57 | -0.58 | 0.74 | 13.48 | -6.16 | -6.37 |
| | 1.84 | 1.01 | 3.63 | 3.45 | -0.10 | 2.78 | 7.24 | -2.81 | -0.17 | 15.53 | -8.65 | -8.30 |
| | 4.79 | -3.10 | 2.59 | 6.94 | -4.67 | 0.63 | 11.34 | -7.88 | -3.72 | 19.25 | -13.17 | -12.00 |
| 8 | 1.22 | 4.51 | 2.49 | 2.56 | 3.50 | 2.24 | 6.01 | 0.85 | 0.17 | 14.48 | -5.55 | -7.74 |
| | 1.60 | 3.71 | 3.00 | 3.14 | 2.53 | 2.37 | 6.93 | -0.40 | -0.40 | 15.61 | -6.95 | -8.81 |
| | 2.80 | 1.74 | 3.15 | 4.71 | 0.25 | 1.77 | 9.02 | -3.10 | -2.08 | 17.82 | -9.68 | -10.97 |
| | 6.61 | -3.37 | 0.59 | 8.93 | -5.19 | -1.65 | 13.57 | -8.78 | -6.33 | 21.84 | -14.71 | -14.96 |

**Supplementary Table S4 | Relative deconvolution error** ($\Delta_{rel}C'_{out} \bullet 100$) for a DAB / HTX / FastRed triple stain modelled with **D65** illumination and non-normalized stain vectors for different input concentrations $c'_{in}$. (data related to Tab. S3) (Rows in fields displays the output values ($c'_{outDAB}$, $c'_{outHTX}$, $c'_{outFastRed}$) for $c'_{inFastRed}$ ={1,2,4,8} (see Fig. S5))

| | $c'_{inDAB}$ = 1 | | | 2 | | | 4 | | | 8 | | |
|---|---|---|---|---|---|---|---|---|---|---|---|---|
| $C'_{inHTX}$ = 1 | 0.2 | -24.6 | 14.5 | 3.8 | -59.7 | 16.1 | 13.8 | -178.8 | -39.8 | 39.0 | -581.4 | -504.4 |
| | 8.6 | -52.9 | 4.9 | 12.4 | -101.4 | -1.2 | 24.6 | -249.3 | -53.6 | 50.0 | -689.7 | -335.4 |
| | 51.4 | -143.2 | -11.6 | 46.1 | -220.3 | -25.9 | 55.8 | -423.9 | -80.6 | 72.7 | -907.8 | -257.5 |
| | 275.3 | -460.3 | -53.8 | 184.8 | -587.6 | -74.5 | 144.1 | -856.9 | -123.1 | 115.1 | -1308.4 | -220.4 |
| 2 | -1.5 | -22.9 | 31.2 | 3.3 | -45.0 | 36.7 | 15.9 | -115.3 | -28.0 | 44.3 | -339.6 | -546.9 |
| | 8.8 | -40.4 | 12.7 | 13.7 | -69.5 | 6.7 | 28.4 | -154.9 | -53.7 | 55.9 | -397.1 | -361.4 |
| | 59.0 | -92.9 | -10.1 | 52.8 | -137.1 | -26.2 | 63.2 | -250.8 | -87.0 | 79.7 | -511.8 | -273.9 |
| | 306.0 | -267.5 | -57.8 | 203.8 | -336.7 | -79.9 | 156.8 | -481.1 | -130.7 | 123.7 | -721.7 | -230.4 |
| 4 | -1.9 | -28.5 | 71.6 | 5.4 | -44.2 | 77.7 | 23.0 | -90.3 | -20.2 | 55.7 | -222.7 | -645.6 |
| | 14.8 | -40.8 | 29.2 | 20.7 | -60.4 | 19.5 | 39.2 | -114.4 | -63.2 | 68.5 | -254.1 | -418.3 |
| | 84.3 | -74.8 | -9.3 | 72.4 | -102.5 | -30.5 | 80.9 | -170.2 | -104.1 | 94.1 | -316.1 | -307.5 |
| | 379.2 | -177.5 | -67.6 | 247.0 | -216.8 | -92.2 | 183.5 | -296.9 | -146.5 | 140.6 | -429.3 | -250.0 |
| 8 | 22.0 | -43.7 | 148.5 | 28.2 | -56.3 | 123.8 | 50.1 | -89.4 | -82.8 | 81.0 | -169.4 | -873.8 |
| | 60.0 | -53.6 | 50.0 | 56.9 | -68.4 | 18.4 | 73.2 | -105.0 | -120.2 | 95.1 | -186.9 | -540.7 |
| | 180.0 | -78.2 | -21.3 | 135.6 | -96.9 | -55.8 | 125.4 | -138.7 | -151.9 | 122.7 | -221.0 | -374.2 |
| | 560.7 | -142.1 | -92.6 | 346.5 | -164.9 | -120.7 | 239.2 | -209.7 | -179.2 | 173.0 | -283.9 | -287.0 |

**Supplementary Table S5 | Deconvolution results** $c'_{out}$ for a DAB / HTX / FastRed triple stain modelled with **RGB LED** illumination and non-normalized stain vectors for different input concentrations $c'_{in}$. (Rows in fields displays the output values ($c'_{outDAB}$, $c'_{outHTX}$, $c'_{outFastRed}$) for $c'_{inFastRed}$ ={1,2,4,8} (see Fig. S5))

| | $c'_{inDAB}$ = 1 | | | 2 | | | 4 | | | 8 | | |
|---|---|---|---|---|---|---|---|---|---|---|---|---|
| $c'_{inHTX}$ = 1 | 0.99 | 0.99 | 1.03 | 2.00 | 0.96 | 1.04 | 4.03 | 0.86 | 1.02 | 8.21 | 0.43 | 0.78 |
| | 1.00 | 0.97 | 2.02 | 2.00 | 0.94 | 2.04 | 4.04 | 0.82 | 2.01 | 8.26 | 0.34 | 1.71 |
| | 1.02 | 0.92 | 3.98 | 2.03 | 0.87 | 3.99 | 4.09 | 0.72 | 3.93 | 8.42 | 0.10 | 3.43 |
| | 1.17 | 0.71 | 7.64 | 2.22 | 0.61 | 7.58 | 4.39 | 0.32 | 7.32 | 9.15 | -0.72 | 5.98 |
| 2 | 0.99 | 1.97 | 1.04 | 1.98 | 1.94 | 1.08 | 4.00 | 1.83 | 1.11 | 8.15 | 1.38 | 0.96 |
| | 0.99 | 1.95 | 2.04 | 1.99 | 1.92 | 2.08 | 4.01 | 1.79 | 2.10 | 8.20 | 1.29 | 1.90 |
| | 1.01 | 1.90 | 4.00 | 2.01 | 1.85 | 4.04 | 4.06 | 1.69 | 4.03 | 8.34 | 1.06 | 3.65 |
| | 1.16 | 1.68 | 7.67 | 2.19 | 1.58 | 7.65 | 4.34 | 1.30 | 7.46 | 9.03 | 0.25 | 6.28 |
| 4 | 0.99 | 3.91 | 1.07 | 1.97 | 3.87 | 1.15 | 3.96 | 3.74 | 1.27 | 8.05 | 3.24 | 1.33 |
| | 0.99 | 3.88 | 2.07 | 1.97 | 3.84 | 2.15 | 3.96 | 3.69 | 2.27 | 8.08 | 3.15 | 2.30 |
| | 1.01 | 3.82 | 4.03 | 2.00 | 3.76 | 4.11 | 4.00 | 3.58 | 4.22 | 8.20 | 2.93 | 4.10 |
| | 1.15 | 3.57 | 7.72 | 2.16 | 3.48 | 7.76 | 4.25 | 3.19 | 7.72 | 8.82 | 2.13 | 6.90 |
| 8 | 1.03 | 7.62 | 1.11 | 1.98 | 7.56 | 1.28 | 3.91 | 7.37 | 1.59 | 7.86 | 6.73 | 2.12 |
| | 1.03 | 7.58 | 2.10 | 1.99 | 7.51 | 2.27 | 3.91 | 7.31 | 2.60 | 7.88 | 6.63 | 3.12 |
| | 1.06 | 7.47 | 4.05 | 2.01 | 7.39 | 4.23 | 3.95 | 7.16 | 4.57 | 7.97 | 6.37 | 5.02 |
| | 1.21 | 7.14 | 7.74 | 2.18 | 7.03 | 7.92 | 4.16 | 6.70 | 8.20 | 8.45 | 5.54 | 8.16 |

**Supplementary Table S6 | Relative deconvolution error** ($\Delta_{rel}c'_{out}$ • 100) for a DAB / HTX / FastRed triple stain modelled with **RGB LED** illumination and non-normalized stain vectors for different input concentrations $c'_{in}$. (data related to Tab. S5) (Rows in fields displays the output values ($c'_{outDAB}$, $c'_{outHTX}$, $c'_{outFastRed}$) for $c'_{inFastRed}$ ={1,2,4,8} (see Fig. S5))

| | $c'_{inDAB}$ = 1 | | | 2 | | | 4 | | | 8 | | |
|---|---|---|---|---|---|---|---|---|---|---|---|---|
| $c'_{inHTX}$ = 1 | -0.6 | -1.3 | 2.5 | -0.2 | -4.0 | 3.8 | 0.6 | -14.5 | 2.3 | 2.6 | -57.4 | -22.3 |
| | -0.4 | -2.8 | 1.1 | 0.0 | -6.2 | 1.8 | 0.9 | -18.3 | 0.7 | 3.3 | -66.4 | -14.7 |
| | 1.7 | -7.6 | -0.5 | 1.4 | -12.5 | -0.3 | 2.2 | -28.5 | -1.7 | 5.3 | -89.6 | -14.3 |
| | 17.3 | -29.0 | -4.5 | 11.0 | -38.7 | -5.2 | 9.9 | -67.5 | -8.6 | 14.4 | -171.9 | -25.3 |
| 2 | -1.1 | -1.5 | 4.3 | -0.8 | -3.0 | 7.7 | 0.0 | -8.6 | 10.7 | 1.9 | -31.0 | -3.9 |
| | -0.8 | -2.3 | 2.1 | -0.6 | -4.2 | 3.9 | 0.3 | -10.6 | 5.1 | 2.5 | -35.4 | -4.9 |
| | 1.1 | -5.0 | 0.1 | 0.6 | -7.6 | 0.9 | 1.4 | -15.7 | 0.8 | 4.3 | -46.9 | -8.7 |
| | 16.0 | -16.0 | -4.1 | 9.7 | -20.8 | -4.4 | 8.5 | -35.1 | -6.8 | 12.9 | -87.3 | -21.4 |
| 4 | -1.1 | -2.4 | 7.1 | -1.4 | -3.3 | 14.9 | -1.0 | -6.6 | 27.0 | 0.6 | -19.0 | 33.3 |
| | -0.8 | -2.9 | 3.5 | -1.3 | -4.1 | 7.5 | -0.9 | -7.7 | 13.6 | 1.0 | -21.2 | 14.8 |
| | 1.1 | -4.6 | 0.7 | -0.2 | -6.0 | 2.8 | 0.0 | -10.5 | 5.5 | 2.5 | -26.9 | 2.5 |
| | 15.4 | -10.7 | -3.5 | 8.2 | -13.1 | -3.0 | 6.3 | -20.3 | -3.5 | 10.2 | -46.7 | -13.7 |
| 8 | 2.7 | -4.7 | 10.6 | -0.9 | -5.5 | 27.5 | -2.3 | -7.9 | 59.4 | -1.7 | -15.8 | 112.4 |
| | 3.4 | -5.3 | 4.9 | -0.7 | -6.2 | 13.7 | -2.1 | -8.7 | 30.2 | -1.5 | -17.1 | 56.2 |
| | 6.2 | -6.6 | 1.2 | 0.7 | -7.6 | 5.9 | -1.3 | -10.5 | 14.4 | -0.4 | -20.4 | 25.6 |
| | 21.4 | -10.7 | -3.2 | 8.9 | -12.2 | -1.0 | 4.0 | -16.3 | 2.5 | 5.7 | -30.8 | 2.0 |

## S1.8: Display of Concentration Values and Gamma Correction

Deconvolution results are usually calculated as floating point numbers. Often they are converted to 8bit integer data for storage and display. This is done by a linear mapping of the relative concentrations $c'_{out}$. The resulting 8bit concentration images are treated as monochrome images or colour look up tables are applied to generate realistic colour impression.

Alternatively, the relative concentrations $c'_{out}$ can be recalculated into intensity values according to the absorption process. For that, we can rewrite Eqn. (S5) (using Eqn. (S17) and (S22)):

$$I(\lambda)=I_0(\lambda)\cdot e^{-A_p(\lambda)\cdot c'} \qquad \text{(S61)}$$

Under the assumption of a linear absorbance formation, Eqn. (S61) allows the calculation of 8bit RGB display values based on the stain vector and the relative concentrations:

$$R = 255 \cdot e^{-A_{Rp} \cdot c' \cdot k_\gamma} \quad \text{(S62)}$$

$$G = 255 \cdot e^{-A_{Gp} \cdot c' \cdot k_\gamma} \quad \text{(S63)}$$

$$B = 255 \cdot e^{-A_{Bp} \cdot c' \cdot k_\gamma} \quad \text{(S64)}$$

An additional gamma correction factor $k_\gamma$ is used to adapt the RGB signal contrast to the diagnostic conditions. This is useful to compensate the non-linear absorption characteristic and the additional non-linear deformation of the contrast curve, caused by the constant factor arising from stain vector normalization (see Eqn. (S35)).

For illustration, we calculate the RGB display values for DAB and HTX with average normalized stain vectors from Fig. 2 (see main document). The resulting RGB colours are displayed in Fig. S6 and S7 for variations of relative concentrations $c'$ and gamma correction value $k_\gamma$. The D65 and the RGB LED stain vectors from Fig. 2 lead to slightly different RGB display colours. The display colours of both stains are realistic and similar to typical sample colours of microscopic specimen[S3]. The effect of the gamma correction $k_\gamma$ is visible. An appropriate gamma value compensates the non-linear signal contrast and leads to the visual perception of linear relation between RGB signal and concentration.

Altogether, this points to a careful interpretation of concentration images. Such images do not necessarily just display the relative stain concentrations as linear or as exponential intensity values. They can contain additional gamma correction and – depending on the actual software implementation – concentration values outside of the image bit depth can be either simply clipped or mapped into the valid range. This is a problem especially for deconvolution errors in form of negative concentration values, because they lead to intensity values above the maximum intensity range (according to Eqn. (S62) to (S64)).

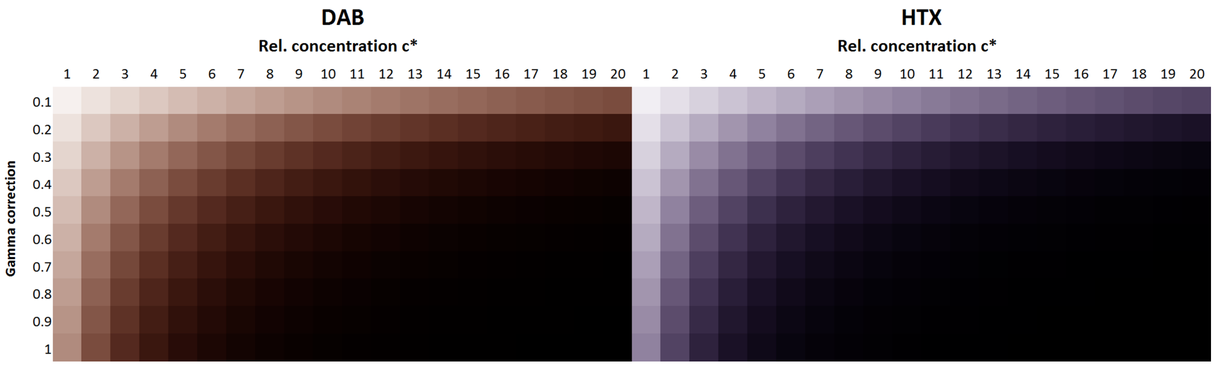


**Supplementary Figure S6 |** ***Reconstructed stain colours*** *for different concentrations of DAB and HTX (normalized stain vectors modelled with* ***D65*** *illumination) and varying gamma correction* $k_\gamma$.

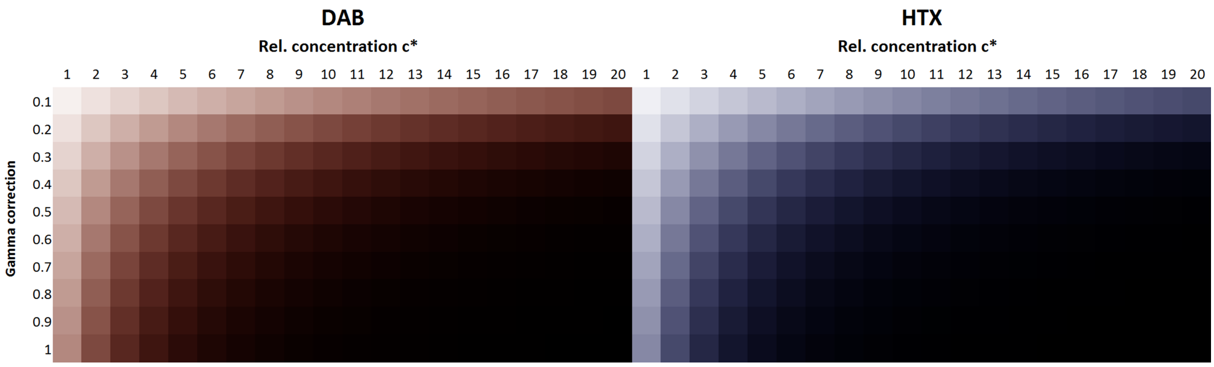


**Supplementary Figure S7 |** ***Reconstructed stain colours*** *for different concentrations of DAB and HTX stains (normalized stain vectors modelled with* ***RGB LED*** *illumination) and varying gamma correction* $k_\gamma$.

## Error Discussion

The quality of imaging based histological and cytological sample analysis is influenced by various aspects. A total error cannot be specified exactly, for example because of the unpredictable variations of sample preparation. Quantification needs stoichiometry, absorbing dyes and intact rather than sectioned objects[S4]. Since measurement also requires specification of its uncertainty, we describe the most critical sources of error and estimate their dimensions. In particular, we discuss influences of sample preparation, imaging and stain properties.

As a precondition, we assume best imaging practice and perfect system setup in terms of optical adjustment, stable and homogeneous illumination and linearity of electronics.

### S1.9: Aspects of IHC Preparation

Beside other staining techniques, immunohistochemistry (IHC) is of special interest in diagnostic pathology. Therefore we particularly review this technique in context of quantitative image analysis.

IHC has changed its status from being "staining" to a qualitative, a semi-quantitative and - more and more - a quantitative target detection method. IHC sample preparation is the most important and at the same time most complex and critical link in IHC quantification. Details of IHC preparation were discussed a long time and huge efforts have been made to standardize the preparation or at least to define application specific guidelines. A large number of publications are addressing preparation, error effects and standardization[S5-S10]. We can summarize that without reference, calibration, reproducibility and error specification there is no measurement, quantification and even qualification.

In IHC, ideally all antigen targets will be found and converted into saturated chromogen precipitate. But sample extraction, pre-treatment, fixation, target retrieval and detection influence the labelling of target signals. This leads to varying relation between chromogen concentration and target signal. Those variations can only be eliminated by standardization of sample processing.

Saturated antigen detection does not necessarily imply stoichiometry of the "staining". Most of the chromogens used in IHC protocols produce non-stoichiometric signals. This is especially the case for both stains examined in our study, DAB and HTX. The "blueing"-step involved in HTX staining cannot be considered stoichiometric. Even DAB staining is not stoichiometric because of its macromolecular super-structure. Quantification errors due to unknown and non-linear antigen-chromogen relationship can only be avoided by standardization and calibration. Especially the latter is difficult to realize because of a missing IHC calibration standard. In addition, the request for biochemical signal saturation can be contradicted by the demand for unsaturated image signals, as will be shown below.

All steps of an IHC protocol can have critical influences. Variations of the protocol might induce unpredictable quantification errors. Thus, IHC interpretation might fall back onto a quantitative level. But nowadays, automated IHC preparation delivers highly reproducible samples. This standardization is important for a reliable correlation between antigen and stain concentration, as found in many studies[24].

The exact characterisation of the effect of IHC variations onto IHC quantification is not our scope. We address the influences of imaging and image analysis. This is essential prior to an analysis of IHC variations.

### S1.10: Imaging Errors

Microscopic imaging is affected by inhomogeneous illumination in the sensor plane due to vignetting and misalignment of optical elements. To ensure correct intensity measurements microscopic images have to be 'flat field' corrected with a background image as reference. Beside this, black level (BL) compensation is necessary. This is done by subtracting the camera noise floor from the image signal. Both, flat field and black level correction are essential in transmitted light microscopy applications.

Quantitative imaging should avoid "signal saturation". In transmitted light microscopy the signal is actually not light intensity but strength of absorption. The minimum camera signal represents the "saturation level". This minimum signal is equivalent to the maximum measurable concentration of a stain. It is determined by the camera noise floor. To prevent signal saturation, camera values should exceed the noise floor plus the shot noise (rule of thumb: 1.5 to 2 times BL).

In digital imaging there are two types of shot noise, the camera shot noise and the photon shot noise. The camera shot noise depends on the specific camera electronics and operating parameters. The photon shot noise is the non-linear noise of the incident light. It is proportional to the square root of the mean number of photon electrons $n_{Signal}$.

For low light levels the total shot noise is mainly defined by the camera electronics. For high light levels it is determined mainly by the photon shot noise. An approximation of the number of total shot noise electrons is given by:

$$n_{noise} \cong \sqrt{n_{signal} + n_{CCD}^2 + n_{AMP}^2} \qquad \text{(S65)}$$

where $n_{CCD}$ is the numbers of sensor shot noise electrons and $n_{AMP}$ is the numbers of amplifier shot noise electrons.

Assuming linear camera characteristics with an electronic conversion factor $G^* = \frac{I_{\max}}{n_{FullWell}}$ we get

$$\Delta I_{noise} \cong \sqrt{G^* \cdot I_{signal} + \Delta I_{CCD}^2 + \Delta I_{AMP}^2} \qquad \text{(S66)}$$

With Eqn. (S66) the total shot noise $\Delta I_{noise}$ can be estimated for camera intensity $I_{signal}$.

For our calculation we assume typical values of an uncooled 8bit camera with full well capacity $n_{FullWell}$=50.000 electrons, sensor shot noise $\Delta I_{CCD}$=1, amplifier shot noise $\Delta I_{AMP}$=1 and maximum camera intensity value $I_{max}$=255. For the entire intensity range of $I_{signal}$=0..255 a nearly constant intensity variation $\Delta I_{noise}$ ~ 1.6 was experimentally calculated. In the following we ignore the shot noise amplification caused by the flat field correction.

Quantization noise is an additional signal uncertainty. It is caused by the analog-to-digital conversion because a whole range of light intensities create identical output values. This quantization noise $\Delta I_{ADC}$ is about a quarter of an intensity digit (or 0.25 LSB - least significant bit).

From total shot noise and quantization noise the average signal noise ΔI can be estimated:

$$\Delta I = \sqrt{\Delta I_{ADC}^2 + \Delta I_{noise}^2} = \sqrt{0.25^2 + 1.6^2} \approx 1.6$$

The above noise model gives a principle description of noise sources and an explanation of their additive characteristics. However, the signal noise estimated here is a ‘best case’ value, only found under ideal imaging conditions. It is close to values typically stated in sensor data sheets. In practical situations, noise values are higher.

Because of the logarithmic transformation of intensity into concentration values (Eqn. (S7)), the resulting concentration uncertainty is non-linear. It can be calculated by

$$\frac{\Delta c}{c} = \frac{\frac{c_1 - c_2}{2}}{\frac{c_1 + c_2}{2}} = \frac{-\frac{1}{2\delta(\lambda)}\ln\left(\frac{I - \Delta I}{I_0}\right) + \frac{1}{2\delta(\lambda)}\ln\left(\frac{I + \Delta I}{I_0}\right)}{-\frac{1}{\delta(\lambda)}\ln\left(\frac{I}{I_0}\right)} \qquad \text{(S67)}$$

$$\frac{\Delta c}{c} = -\frac{1}{2}\frac{\ln\left(\frac{I + \Delta I}{I - \Delta I}\right)}{\ln\left(\frac{I}{I_0}\right)} \qquad \text{(S68)}$$

With this equation, the relative concentration uncertainty due to shot noise and quantization noise can be calculated.

In Fig. S8 the concentration error is displayed for the image intensity $I=I_{cam}$–BL, a black level BL=5, a maximum signal $I_0$=255–BL and a signal noise ΔI=±0.8 for the intensity range $I_{cam}$=0..255. The graph demonstrates that concentration values are accurate only in the mid intensity range. The relative concentration error rises up to 20% for low intensities

and up to 80% for high intensities. For high concentrations this error is determined by the camera shot noise. It can be reduced by camera cooling. For low concentrations it is determined by the photon shot noise and cannot be eliminated.

According to this result, low as well as high concentration values are affected by the quantisation error. Depending on the application requirements, image elements with intensities close to BL respectively to $I_{max}$ should be rejected.

This quantization error is a statistical error originating from logarithmic conversion of the signal noise. It is independent of the deconvolution error and small compared to other influences. However, it contributes to the total measurement error by a few percent.

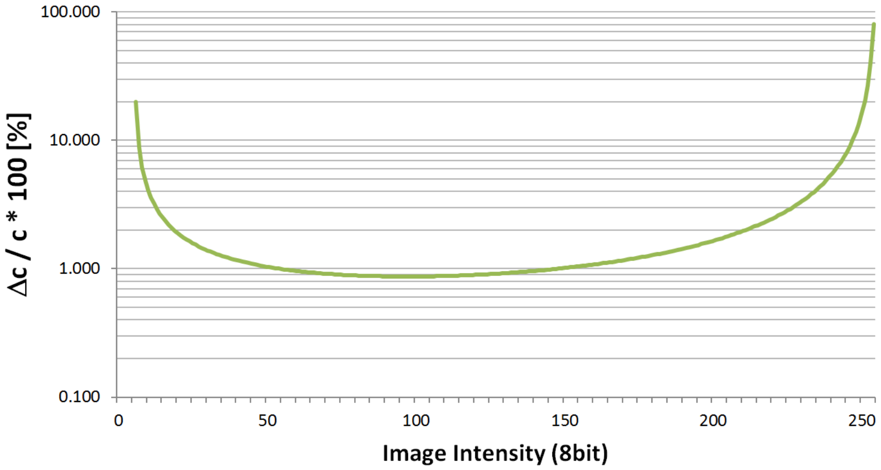


**Supplementary Figure S8 |** ***Relative concentration error*** *induced by logarithmic conversion of image noise ('best case' scenario with low image noise $\Delta I=\pm 0.8$ LSB)*

## S1.11: DAB Absorption and Scattering

In biomedical light microscopy the image contrast is formed by an interaction between the incident light and the atoms, molecules and the structures of tissue and cell samples. The electromagnetic field of the photons is scattered by the objects of matter [S11-S17]. The macroscopic effects of these alterations can be – even in a complex way - described by the Maxwell equations.

Inelastic scattering processes, such as Raman scattering and fluorescence, lead to light emission with wavelengths different from the incident light. In transmitted light imaging they can be neglected due to their vanishing intensity. Under standard conditions an elastic scattering is the predominant process. It can be divided into resonance and non-resonance scattering. Elastic scattering has the fundamental characteristics of an undetermined scattering direction, which for homogeneous and dense media leads to an interference induced net forward scattering. Therefore, the incoming light seems to simply pass the homogeneous, dense medium. Under certain conditions, the passing light is attenuated by dissipative resonance absorption. This absorption can be considered as special type of scattering, because it is initiated by the same physical interaction of photons and matter.

That said, the well-known effect of one of the most important chromogens in diagnostic imaging, 3'-3'diaminobenzidin (DAB), being "not a true absorber"[S4, S18] can be treated more specific. DAB molecules are good absorbers in the visible wavelength region due their conjugated electron system. Besides that, it is known that the brown DAB staining is also "created" by the polymeric, amorphous, non-droplet structure of the DAB macromolecules[S4, S19, S20]. The assumption of a homogeneous substrate is not valid for small, condensed particles and the effect of net forward scattering is lost. DAB particles are contributing to the light extinction in an additional way, different from resonance absorption described by Lambert-Beer. DAB is not a 'pure' absorber. DAB absorbs and scatters. The scatter characteristic depends on the DAB particle size, form and distribution which are all varying with the chemical properties of the DAB substance and the preparation conditions[S4].

Both, absorption and scattering, lead to a concentration dependent alteration of the spectral extinction. The concentration dependency of the scattering effect cannot be linearized by logarithmic transformation like the absorption effect (Eqn.

(S33)). The scattering is the source of the non-linear behaviour of the DAB stain. Bernardo et al.[22] cited “However, using DAB as a chromogen is problematic because a linear relationship between the amount of antigen and staining intensity exists only at low levels of the latter”[S21] and “Image analysis systems that assess the amount of staining by measuring absorption can yield inaccurate readings because of the non- linearity of signal at higher levels of antigen”[S5]. DAB is a good scatterer for electron and darkfield microscopy, but it is not well-suited for quantitative photometry.

So far, we have treated DAB staining in our study as pure absorber because (i) the deconvolution error could as well be studied with arbitrary absorption spectra and (ii) we aimed to determine the undistorted deconvolution error and thereby the maximum achievable accuracy of CD measurements.

However, to give a comprehensive view we add a scattering component into our numeric model. Thereby we can compare deconvolution error and ‘scatter error’.

## S1.12: Simulation of DAB Scattering

Due to the particle character of the DAB reaction product the Lambert-Beer equation (Eqn. (S2)), which assumes small stain concentrations and no interaction between the absorbing molecules, is incomplete for DAB. Photometric quantification and other methods based on light excitation cannot simply handle such ‘particle stains’ as pure absorbing molecules. The light scattering has to be incorporated into the extinction process.

For an estimation of the influence of light scattering we assume a DAB particle size far below the visible wavelength range. This seems plausible because of the applicability of DAB in electron microscopy. Therefore we can – as a first order approximation – assume a Rayleigh characteristic of the scattering.

We can extend Eqn. (S2) to

$$I(\lambda) = I_0(\lambda) \cdot e^{-(a(\lambda)+h(\lambda,N))\cdot s} \tag{S69}$$

Equation (S69) describes the extinction as function of absorption and scattering.

Herein, the Rayleigh scattering coefficient h(λ,N) depends on the wavelength λ, the wavelength specific refraction index n(λ) and the number of molecules per volume N.

$$h(\lambda, N) = \frac{8\pi^3}{3N\lambda^4}\left(n^2(\lambda)-1\right)^2 \tag{S70}$$

With the proportionality $\left(n^2(\lambda)-1\right)^2 \sim N^2$ from the theory of dispersion[S12] and with the assumption of normal descending dispersion without anomalies, we can neglect the exact value of n(λ) compared to the $1/\lambda^4$ influence and can approximate the scattering as a function of wavelength λ and relative concentration c’ with

$$h(\lambda, c') \sim \frac{k_1 \cdot \lambda_{\min}^4 \cdot c'^{k_2}}{\lambda^4} \tag{S71}$$

This approximation can be included in our model by modifying Eqn. (S5) (using Eqn. (S17) and (S22)):

$$I(\lambda) = I_0(\lambda) \cdot e^{-\left(A_p(\lambda)+h(\lambda,c')\right)\cdot c'} \tag{S72}$$

In this approach, the scatter contribution and the concentration dependency of the scattering can be controlled by the parameters $k_1$ and $k_2$.

Based on this extended model, we calculated the spectral extinction of ‘scattering DAB’ (sDAB) as well as the deconvolution error of sDAB/HTX double staining.

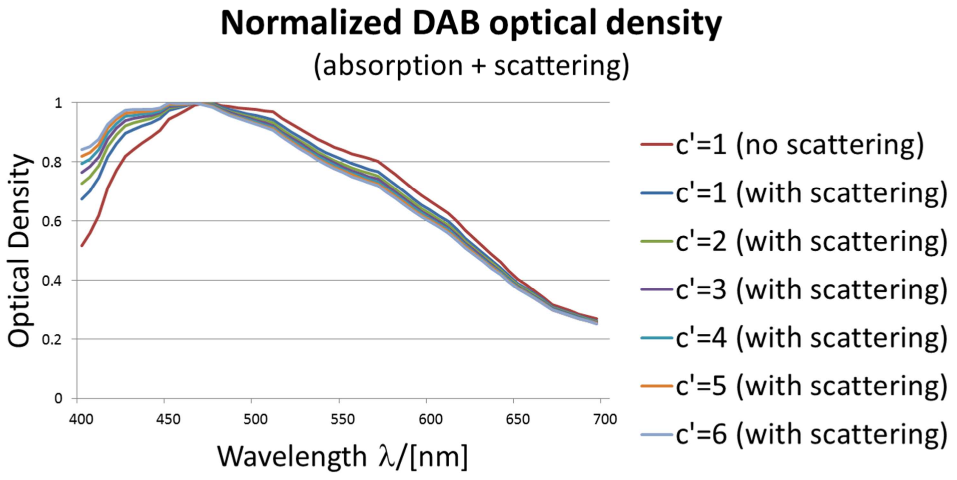


**Supplementary Figure S9 |** ***Optical density of sDAB*** *for various concentrations*

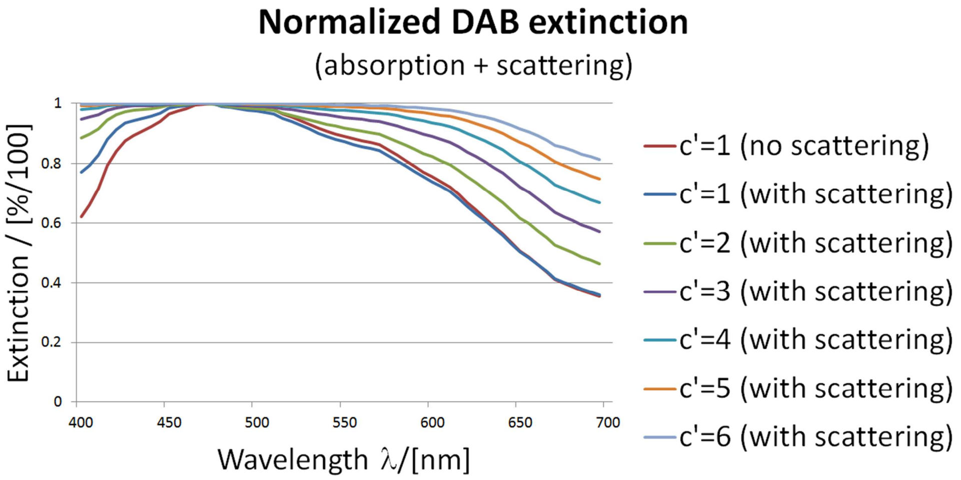


**Supplementary Figure S10 |** ***Extinction of sDAB*** *for various concentrations*

The spectra shown in Fig. S9 and S10 are calculated with $k_1 = 0.2$, $k_2 = 0.5$. These values are chosen by assuming an optical density ratio from scattering and absorption of about 1/2 (for c'=1 and $\lambda = \lambda_{min}$) and an increase of scattering with $\sqrt{c'}$. Furthermore, it is assumed that the optical density values obtained from [sd-3] already include scattering. Therefore, the pure absorption spectra are calculated by subtracting the scattering portion from the original spectral values (with c'=1; $k_1$, $k_2$ as state above). Of course, these arbitrary values of $k_1$ and $k_2$ have to be used as long as no spectral reference data is available.

Figure S9 displays the normalized optical density and demonstrates the effect of scattering for varying DAB concentration. With increasing DAB concentration, the optical density increases for short wavelengths and decreased for long wavelengths because of the signal normalisation. Note, without scattering this normalized optical density spectra would be independent of the DAB concentration per definition.

The absorption spectra in Fig. S10 increases toward saturation with increasing DAB concentration. Due to scattering, the rise of the absorption is stronger for short wavelengths than for long wavelengths. The overall increase of the spectra is not an indicator for scattering as can be seen by calculating these spectra without scattering component.

These spectral results are meaningful and plausible and reflect the behaviour of stains comprising absorption and scatter characteristics.

With the above parameters ($k_1 = 0.2$, $k_2 = 0.5$) we have modelled the deconvolution of sDAB/HTX double staining.

Fig. S11 and S12 display the results for wideband D65 illumination and narrow band RGB LED illumination. Both figures are created analogue to Fig. 3 and 4 in the main document.

The average values of the relative errors $|\Delta c^*_{out}|$ from Fig. S11 and Fig. S12 for the value range of $c^*_{in} = \{1, 2, 4, 8\}$ are:

$$\overline{\left|\Delta c^*_{outDAB}\right|}_{D65} = 69.4\ \% \qquad \overline{\left|\Delta c^*_{outHTX}\right|}_{D65} = 107.1\ \%$$

$$\overline{\left|\Delta c^*_{outDAB}\right|}_{RGBLED} = 13.3\ \% \qquad \overline{\left|\Delta c^*_{outHTX}\right|}_{RGBLED} = 16.3\ \%$$

**a** Deconvolution Results $c^*_{outDAB}$

| $c^*_{inHTX}$ \ $c^*_{inDAB}$ | 0.1 | 1 | 2 | 4 | 8 |
|---|---|---|---|---|---|
| 0.1 | 0.08 | 0.97 | 2.13 | 4.85 | 11.85 |
| 1 | 0.09 | 0.98 | 2.15 | 4.97 | 12.52 |
| 2 | 0.15 | 1.05 | 2.24 | 5.21 | 13.41 |
| 4 | 0.47 | 1.43 | 2.73 | 6.11 | 15.48 |
| 8 | 2.18 | 3.42 | 5.12 | 9.36 | 19.93 |

**b** Deconvolution Results $c^*_{outHTX}$

| $c^*_{inHTX}$ \ $c^*_{inDAB}$ | 0.1 | 1 | 2 | 4 | 8 |
|---|---|---|---|---|---|
| 0.1 | 0.11 | 0.12 | 0.01 | -0.60 | -3.72 |
| 1 | 0.99 | 1.00 | 0.87 | 0.14 | -3.74 |
| 2 | 1.88 | 1.87 | 1.70 | 0.76 | -4.00 |
| 4 | 3.30 | 3.20 | 2.86 | 1.30 | -5.06 |
| 8 | 4.37 | 3.83 | 2.89 | 0.05 | -7.99 |

**c** Rel. Deconvolution Error $|c^*_{outDAB}-c^*_{inDAB}|/c^*_{inDAB}*100$

| $c^*_{inHTX}$ \ $c^*_{inDAB}$ | 0.1 | 1 | 2 | 4 | 8 |
|---|---|---|---|---|---|
| 0.1 | 15.9 | 2.5 | 6.3 | 21.2 | 48.1 |
| 1 | 5.8 | 1.5 | 7.5 | 24.2 | 56.6 |
| 2 | 51.5 | 5.0 | 12.1 | 30.1 | 67.6 |
| 4 | 370.1 | 42.6 | 36.6 | 52.9 | 93.4 |
| 8 | 2081.3 | 241.7 | 155.9 | 134.0 | 149.1 |

**d** Rel. Deconvolution Error $|c^*_{outHTX}-c^*_{inHTX}|/c^*_{inHTX}*100$

| $c^*_{inHTX}$ \ $c^*_{inDAB}$ | 0.1 | 1 | 2 | 4 | 8 |
|---|---|---|---|---|---|
| 0.1 | 10.8 | 19.0 | 89.2 | 696.7 | 3815.8 |
| 1 | 1.1 | 0.3 | 12.9 | 86.5 | 473.6 |
| 2 | 6.1 | 6.5 | 14.8 | 61.9 | 299.8 |
| 4 | 17.6 | 20.0 | 28.5 | 67.4 | 226.4 |
| 8 | 45.3 | 52.1 | 63.8 | 99.4 | 199.8 |

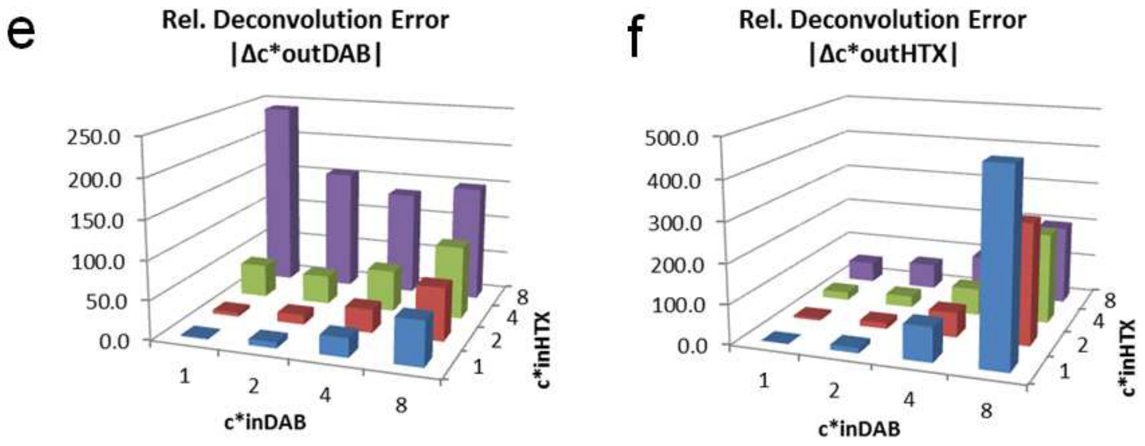


**Supplementary Figure S11 |** ***Deconvolution of sDAB/HTX*** *double stain modelled with* ***D65*** *illumination and SonyICX285AQ color CCD sensor. The model comprises* ***scattering****. Shown are concentration results $c^*_{out}$ for DAB (a) and HTX (b) and relative deconvolution errors for DAB (c, e) and HTX (d, f). The modelling was based on normalised stain vectors and was calculated for different input concentrations $c^*_{in}$. The deconvolution was performed in the B/G plane projection.*

**a** Deconvolution Results $c^*_{outDAB}$

| $c^*_{inHTX}$ \ $c^*_{inDAB}$ | 0.1 | 1 | 2 | 4 | 8 |
|---|---|---|---|---|---|
| 0.1 | 0.09 | 0.98 | 2.08 | 4.50 | 10.05 |
| 1 | 0.09 | 0.97 | 2.06 | 4.47 | 9.99 |
| 2 | 0.10 | 0.98 | 2.06 | 4.45 | 9.94 |
| 4 | 0.17 | 1.03 | 2.09 | 4.45 | 9.85 |
| 8 | 0.48 | 1.31 | 2.34 | 4.62 | 9.84 |

**b** Deconvolution Results $c^*_{outHTX}$

| $c^*_{inHTX}$ \ $c^*_{inDAB}$ | 0.1 | 1 | 2 | 4 | 8 |
|---|---|---|---|---|---|
| 0.1 | 0.11 | 0.11 | 0.06 | -0.17 | -1.11 |
| 1 | 1.00 | 1.02 | 0.98 | 0.78 | -0.10 |
| 2 | 1.97 | 2.00 | 1.98 | 1.81 | 1.01 |
| 4 | 3.84 | 3.89 | 3.90 | 3.80 | 3.16 |
| 8 | 7.22 | 7.32 | 7.39 | 7.42 | 7.12 |

**c** Rel. Deconvolution Error $|c^*_{outDAB}-c^*_{inDAB}|/c^*_{inDAB}*100$

| $c^*_{inHTX}$ \ $c^*_{inDAB}$ | 0.1 | 1 | 2 | 4 | 8 |
|---|---|---|---|---|---|
| 0.1 | 12.0 | 2.2 | 3.8 | 12.5 | 25.6 |
| 1 | 9.9 | 2.7 | 3.2 | 11.8 | 24.9 |
| 2 | 3.3 | 2.1 | 3.0 | 11.3 | 24.2 |
| 4 | 68.9 | 3.0 | 4.7 | 11.2 | 23.1 |
| 8 | 379.7 | 31.4 | 17.2 | 15.5 | 22.9 |

**d** Rel. Deconvolution Error $|c^*_{outHTX}-c^*_{inHTX}|/c^*_{inHTX}*100$

| $c^*_{inHTX}$ \ $c^*_{inDAB}$ | 0.1 | 1 | 2 | 4 | 8 |
|---|---|---|---|---|---|
| 0.1 | 6.5 | 13.3 | 36.0 | 269.2 | 1213.3 |
| 1 | 0.1 | 2.0 | 1.6 | 21.9 | 110.1 |
| 2 | 1.4 | 0.2 | 0.8 | 9.3 | 49.6 |
| 4 | 4.1 | 2.7 | 2.5 | 4.9 | 21.1 |
| 8 | 9.8 | 8.5 | 7.7 | 7.2 | 11.0 |

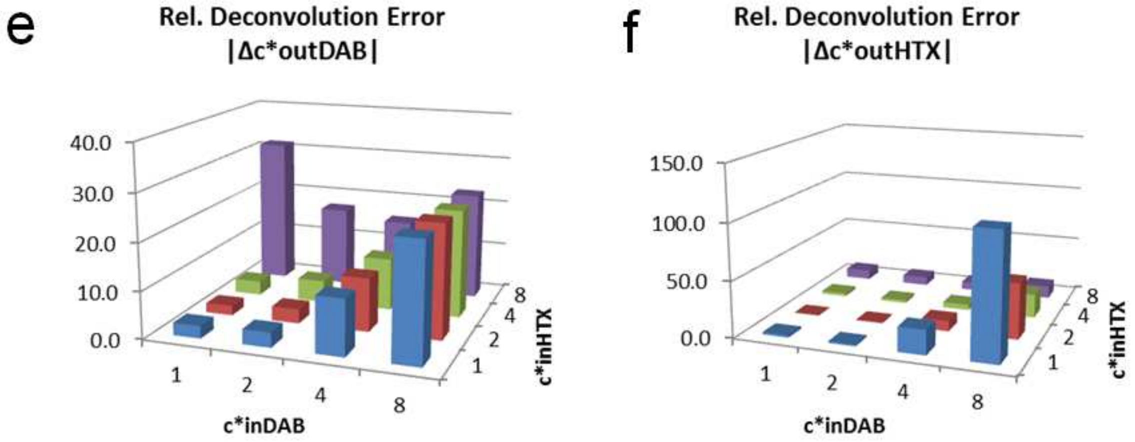


**Supplementary Figure S12 | Deconvolution** ***of sDAB/HTX*** *double stain modelled with* ***RGB LED*** *illumination and SonyICX285AL monochrome CCD sensor. The model comprises* ***scattering****. Shown are concentration results* $c^*_{out}$ *for DAB (a) and HTX (b) and relative deconvolution errors for DAB (c, e) and HTX (d, f). The modelling was based on normalised stain vectors and was calculated for different input concentrations* $c^*_{in}$*. The deconvolution was performed in the B/G plane projection.*

Both values $\overline{|\Delta c^*_{out}|}_{D65}$ are by 50% increased compared to the values in the main document. The values $\overline{|\Delta c^*_{out}|}_{RGBLED}$ are by 200% respectively 500% increased compared to the values in the main document.

This increase can be interpreted as scattering effect. For D65, the additional error induced by DAB scattering is smaller than the deconvolution error. For RGB LED, the quasi monochromatic characteristic is diminished by DAB scattering. The absolute error induced by scattering is smaller for RGB LED. The RGB LED is still the more “accurate illumination”.

DAB scattering mostly affects the stain mixtures with medium and high DAB concentrations. Therefore, we compare error values $\overline{|\Delta c^*_{out}|}$ averaged in the range $c^*_{inDAB} = \{4, 8\}$ and $c^*_{inHTX} = \{1,2,4, 8\}$. According to Fig. S11 and Fig. 3, the scattering induced error for D65 and medium/high DAB concentration is about the same size as the deconvolution error. We conclude that the scattering error is not a predominant effect. When imaging is based on wideband spectra, the deconvolution error is not negligible in relation to the scatter error – even not for moderate DAB concentrations.

For narrow band illumination, the scattering effect dominates the error. The small RGB LED deconvolution error is irrelevant compared to the scatter error.

The deconvolution error, the quantization error and the scattering error increase with the DAB concentration. Therefore, care must be taken interpreting deconvolution results from high stain concentrations. This should not mislead to the assumption that errors are negligible for small and medium concentrations. Medium DAB concentrations of $c^*_{DAB} = 4$ are commonly found in IHC measurements (compare stain colour in Fig. S6 and S7). According to Fig. S11, the D65 errors for $c^*_{DAB}=4$ range from 25% up to 85%. In our opinion, errors of this dimension should be included into measurement interpretation – especially because they are not statistical errors, but distinct methodical aberrances.

The aim of our simple scattering model was to estimate the dimension of the scatter influence. It was not our intent to analyse DAB scattering in all details. However, the approach is efficient and useful. With a better understanding of the scatter characteristics and the availability of reference data, this model could be optimized, approved and integrated into stain separation techniques.

## Appendix

### References


[S1] Wu Q., Merchant F. A. & Castleman K. R. *Microscope Image Processing* (Elsevier Academic Press, 2008).

[S2] Burger W. & Burge M. *Digital Image Processing – An Algorithmic Introduction using Java* (Springer Science+Business Media, 2010).

[S3] van der Loos C. M. (2010). Chromogens in multiple immunohistochemical staining used for visual assessment and spectral imaging: the colorful future. *J. Histotechnol.* **33,** 31-40 (2010).

[S4] Suvarna, K. S. *Bancroft's Theory and Practice of Histological Techniques* (Elsevier Health Sciences, 2013).

[S5] Walker, R. A. Quantification of immunohistochemistry - issues concerning methods, utility and semiquantitative assessment I. *Histopathology* **49**, 406-410 (2006).

[S6] Taylor, C. R. & Levenson, R. M. Quantification of immunohistochemistry -- issues concerning methods, utility and semiquantitative assessment II. *Histopathology* **49**, 411-424 (2006).

[S7] Mighell, A., Hume, W. & Robinson, P. A. An overview of the complexities and subtleties of immunohistochemistry. *Oral Dis.* **4**, 217-223 (1998).

[S8] Pasquale, C. et al. A user's guide for avoiding errors in absorbance image cytometry: a review with original experimental observations. *Histochem.* **26**, 1-19 (1994).

[S9] Yaziji, H. & Barry, T. Diagnostic Immunohistochemistry: what can go wrong?. *Adv. Anat. Pathol.* **13**, 238-246 (2006).

[S10] Seidal, T., Balaton, A. J. & Battifora, H. Interpretation and quantification of immunostains. *Am. J. Surg. Pathol.* **25**, 1204-1207 (2001).

[S11] Hecht, E. *Optics 4th edn* (Addison-Wesley, 2002).

[S12] Bergmann, Schaefer & Niedrig *Lehrbuch der Experimentalphysik, Band 3: Optik Aufl. 10* (de Gruyter Verlag, 2004).

[S13] Vo-Dinh, T., *Biomedical Photonics Handbook* (CRC press, 2014).

[S14] Mourant, J. R. et al. Mechanisms of light scattering from biological cells relevant to noninvasive optical-tissue diagnostics. *Appl. Optics* **37**, 3586-3593 (1998).

[S15] Mishchenko, M. I., Larry D. T. & Lacis, A. A. *Scattering, Absorption, and Emission of Light by Small Particles* (Cambridge University Press, 2002)

[S16] Popp, A. K., Valentine, M. T., Kaplan, P. D. & Weitz, D. A. Microscopic origin of light scattering in tissue. *Appl. Optics* Vol. **42**, (2003).

[S17] Laughney, A. M. *Localized spectroscopic imaging for the detection and discrimination of surgical breast tissue pathologies*. (Thesis) Dartmouth College, 2012.

[S18] van der Loos, C. M. Multiple immunoenzyme staining: methods and visualizations for the observation with spectral imaging. *J. Histochem. Cytochem.* **56**, 313-328 (2008).

[S19] Graham, R. C. & Karnovsky, M. J. The early stages of absorption of injected horseradish peroxidase in the proximal tubules of mouse kidney: ultrastructural cytochemistry by a new technique. *J. Histochem. Cytochem.* **14**, 291-302 (1966).

[S20] Seligman, A. M., Karnovsky, M. J., Wasserkrug, H. L. & Hanker, J. S. Nondroplet ultrastructural demonstration of cytochrome oxidase activity with a polymerizing osmiophilic reagent, diaminobenzidine (DAB). *J. Cell Biol.* **38**, 1-14 (1968).

[S21] Fritz, P., Wu, X., Tuczek, H., Multhaupt, H. & Schwarzmann, P. Quantitation in immunohistochemistry. A research method or a diagnostic tool in surgical pathology?. *Pathologica* **87**, 300–309 (1995).

## Symbols

| | |
|---|---|
| $\alpha,\beta,\gamma$ | Vector slope in plane projections |
| $\alpha\ (\lambda)$ | Spectral absorption coefficient |
| $\delta(\lambda)$ | Spectral molar optical density |
| $\tau(\lambda)$ | Spectral transmission of a filter / stain |
| $\varphi_\Delta$ | Angle between stain vectors |
| $\chi(\lambda)$ | Molar absorption coefficient |
| $A_R$, $A_G$, $A_B$ | Absorbance (red, green, blue spectral band) |
| $A(\lambda)$ | Spectral absorbance |
| BL | Black level / noise floor |
| c | Concentration |
| $c'$ | Relative concentration |
| $c^*$ | Normalized relative concentration |
| G* | Electronic conversion factor |
| $h(\lambda, N)$ | Rayleigh scattering coefficient |
| $h(\lambda, c')$ | Approximated Rayleigh scattering coefficient |
| I | Image intensity value |
| $I(\lambda)$ | Filtered spectral light intensity function |
| $I_0(\lambda)$ | Spectral light intensity function |
| $I_{cam}$ | Camera intensity value |
| $I_{max}$ | Maximum signal |
| $I_{rel}(\lambda)$ | Relative spectral light intensity function |
| $\Delta I$ | Signal uncertainty / signal noise |
| $\Delta I_{ADC}$ | Signal quantization noise |
| $\Delta I_{AMP}$ | Signal amplifier shot noise |
| $\Delta I_{CCD}$ | Signal sensor shot noise |
| $\Delta I_{noise}$ | Signal total shot noise |
| $k_1$, $k_2$ | Parameters controlling the contribution and the concentration dependency of the DAB scattering |
| $k_{CCD}$ | CCD factor (linear signal amplification) |

| | |
|---|---|
| $k_\gamma$ | Gamma correction factor |
| $n(\lambda)$ | Spectral refraction index |
| $n_{AMP}$ | Numbers of amplifier shot noise electrons |
| $n_{CCD}$ | Numbers of sensor shot noise electrons |
| $n_{FullWell}$ | Number of full well capacity electrons |
| $n_{noise}$ | Number of total shot noise electrons |
| $n_{Signal}$ | Number of photon electrons |
| N | Number of molecules per volume |
| R, G, B | Linear RGB colour display values |
| R', G', B' | Nonlinear sRGB colour display values |
| s | Thickness of filter / sample |
| $s_R(\lambda)$, $s_G(\lambda)$, $s_B(\lambda)$ | Spectral sensitivity of a RGB colour camera |
| $V_R$, $V_G$, $V_B$ | RGB camera signals of a stained sample |
| $V_{0R}$, $V_{0G}$, $V_{0B}$ | Maximum RGB camera signals |
| $V'_R$, $V'_G$, $V'_B$ | Modelled RGB camera values |
| $V'_{0R}$, $V'_{0G}$, $V'_{0B}$ | Maximum modelled RGB camera values |
| X, Y, Z | CIE XYZ colour coordinates |
| $\bar{x}(\lambda), \bar{y}(\lambda), \bar{z}(\lambda)$ | Spectral sensitivity of a norm observer |

## Indices

| | |
|---|---|
| 1, 2, 3 .. i | Indices of stains |
| a | Index of averaged values |
| k, l, m .. j | Indices of spectral wavelength |
| n | Index of normalised values |
| p | Index of pure stains |
| R, G, B | Indices of red, green, blue spectral band |
| D65 | Index of D65 standard illumination |
| RGB LED | Index of RGB LED illumination |
| DAB | Index of DAB stain |
| HTX | Index of Hematoxylin stain |
| FastRed | Index of FastRed stain |
| in | Index of input concentrations |
| out | Index of output concentrations |
